# How Should the Impact of Different Presentations of Treatment Effects on Patient Choice Be Evaluated? A Pilot Randomized Trial

**DOI:** 10.1371/journal.pone.0003693

**Published:** 2008-11-24

**Authors:** Cheryl Carling, Doris Tove Kristoffersen, Jeph Herrin, Shaun Treweek, Andrew D. Oxman, Holger Schünemann, Elie A. Akl, Victor Montori

**Affiliations:** 1 Norwegian Knowledge Centre for the Health Services, Oslo, Norway; 2 Department of Medicine, Yale University School of Medicine, New Haven, Connecticut, United States of America; 3 Clinical Research and INFORMAtion Translation Unit, and Department of Epidemiology, Italian National Cancer Institute Regina Elena, Rome, Italy; 4 Department of Medicine, State University of New York at Buffalo, Buffalo, New York, United States of America; 5 Knowledge and Encounter Research Unit, Division of Endocrinology and Internal Medicine, Mayo Clinic College of Medicine, Rochester, Minnesota, United States of America; Cardiff University, United Kingdom

## Abstract

**Background:**

Different presentations of treatment effects can affect decisions. However, previous studies have not evaluated which presentations best help people make decisions that are consistent with their own values. We undertook a pilot study to compare different methods for doing this.

**Methods and Findings:**

We conducted an Internet-based randomized trial comparing summary statistics for communicating the effects of statins on the risk of coronary heart disease (CHD). Participants rated the relative importance of treatment consequences using visual analogue scales (VAS) and category rating scales (CRS) with five response options. We randomized participants to either VAS or CRS first and to one of six summary statistics: relative risk reduction (RRR) and five absolute measures of effect: absolute risk reduction, number needed to treat, event rates, tablets needed to take, and natural frequencies (whole numbers). We used logistic regression to determine the association between participants' elicited values and treatment choices. 770 participants age 18 or over and literate in English completed the study. In all, 13% in the VAS-first group failed to complete their VAS rating, while 9% of the CRS-first group failed to complete their scoring (p = 0.03). Different ways of weighting the elicited values had little impact on the analyses comparing the different presentations. Most (51%) preferred the RRR compared to the other five summary statistics (1% to 25%, p = 0.074). However, decisions in the group presented the RRR deviated substantially from those made in the other five groups. The odds of participants in the RRR group deciding to take statins were 3.1 to 5.8 times that of those in the other groups across a wide range of values (p = 0.0007). Participants with a scientific background, who were more numerate or had more years of education were more likely to decide not to take statins.

**Conclusions:**

Internet-based trials comparing different presentations of treatment effects are feasible, but recruiting participants is a major challenge. Despite a slightly higher response rate for CRS, VAS is preferable to avoid approximation of a continuous variable. Although most participants preferred the RRR, participants shown the RRR were more likely to decide to take statins regardless of their values compared with participants who were shown any of the five other summary statistics.

**Trial Registration:**

Controlled-Trials.com ISRCTN85194921

## Introduction

There is a large literature on risk communication, including how different presentations of risk influence understanding, perceptions and decisions; and how information about risks is used in decisions [1]–[12]. Systematic reviews have found that how information about the effects of health care is presented impacts on how that information is perceived and hypothetical decisions, although the impact on real world decisions is less certain [6]–[11]. Differences in presentations include positive versus negative framing, different summary statistics (including relative and absolute measures of effect), and different formats (numeric, verbal and graphical) [5]–[11]. One of the most consistent findings is that presenting a “relative risk reduction” (RRR) as compared to an “absolute risk reduction” (ARR) or the “number needed to treat” (NNT) to express a treatment effect results in more individuals perceiving the treatment effect to be large and more decisions in favour of an intervention, although the magnitude of the impact varies across different studies [5], [10], [11]. However, no previous studies have evaluated which summary statistics best help people to make decisions that are consistent with their own values. For example, although the RRR is more persuasive than the ARR and NNT, this does not necessarily mean that it is better or worse in terms of helping people make decisions that are consistent with their values.

“Values” here refers to the relative importance of the desirable and undesirable effects of an intervention. Different people have different values and these affect the decisions that they make. For example, anticoagulation therapy reduces the risk of stroke and increases the risk of serious gastrointestinal bleeding in patients with atrial fibrillation. The relative importance of a stroke and serious gastrointestinal bleeding varies widely (among both physicians and patients) and these different values lead to different recommendations and decisions about whether to use anticoagulants [13].

Various models of decision making in health care stress the importance of incorporating patients' values for the possible consequences of alternative interventions into a decision [14], [15]. The consistency of a health care decision with the patient's values, along with various emotive, cognitive, and behavioural outcomes, has been used to evaluate the quality of risk communication in patient decision aids or between health care professionals and patients [15]–[17]. For example, in 34 trials of decision aids for screening decisions, two of the four trials that measured agreement between values and choices found an improvement [18].

According to the normative concept of expected utility maximization [19], derived from the expected utility model of Daniel Bernoulli, people should choose the option that gives the highest expected utility_._ The utility (i.e. preference for or desirability) of outcomes, such as different health states, is usually expressed as a number ranging from zero to one, with death having a value of zero and a fully healthy life having a value of one [20], [21].

Expected utility theory has been questioned for a number of reasons, which include problems with how utilities are measured and observations that people often do not, in fact, choose to maximize their utilities [22]–[26]. Nonetheless, it can still be argued that as the expected utility for a decision, e.g. taking statin therapy, increases, one would expect that, on average, increasing proportions of people would choose to take the therapy if they were well informed. This argument does not depend on every individual choosing to maximize his or her utilities. Some people may make decisions based on other factors and it is difficult to accurately measure people's utilities. Nonetheless, amongst patients presented with the same choice options with similar risks, one would expect some degree of correlation between the values that individuals attach to the desirable and undesirable consequences of a decision such as taking medication and the likelihood that they would decide to take the medication. In other words, one would expect that people for whom the benefits of taking medication were less important and the downsides more important would be less likely, on average, to decide to take medication than people for whom the benefits were more important and the downsides were less important.

Several methods are used for eliciting the values that a person places on health outcomes or other consequences of health care decisions [27], [28]. The three most commonly used methods that generate a utility are the time trade-off, rating scales such as visual analogue scales (VAS) and category rating scales (CRS), and the standard gamble (SG), in that order [29]. The CRS is conceptually a linear scale divided into evenly demarcated sections or “categories”, thus forming a “category rating scale” [30]. The standard gamble, which has been criticized because it is difficult to explain to patients who do not find it intuitive [31], and the time trade-off require interviews to administer [32], whereas the VAS and CRS do not.

We report here the results of a pilot study of the Health Information Project: Presentation Online (HIPPO). The goal of the HIPPO project was to compare different ways of presenting information about the effects of health care in order to determine which presentations best help people to make decisions that are consistent with their own values. The objectives of this pilot study were to investigate the feasibility of conducting Internet-based randomized trials comparing different risk reduction presentations; to compare two methods (VAS and CRS) of eliciting values (i.e. the relative importance of the desirable and undesirable consequences of a decision); to explore approaches to combining the elicited values to calculate a total value (“relative importance score”); and to generate hypotheses and calculate sample size for a confirmatory study comparing six summary statistics for communicating evidence of reduced risk of coronary heart disease (CHD) with statin therapy for high cholesterol.

## Methods

The protocol and CONSORT checklist for this study are available as supporting information; see Protocol S1 and CONSORT checklist S1. For a facsimile of this study's Internet site see Protocol S2.

**Figure 1:**
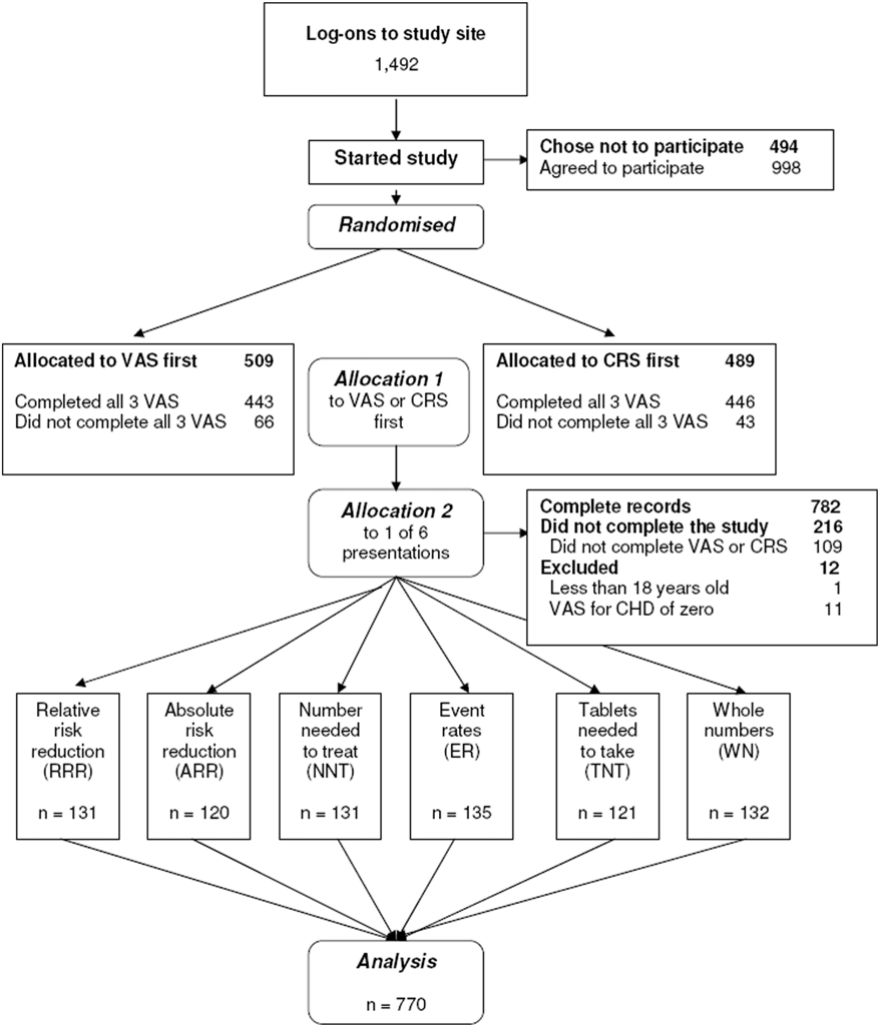
Flowchart

**Figure 2:**
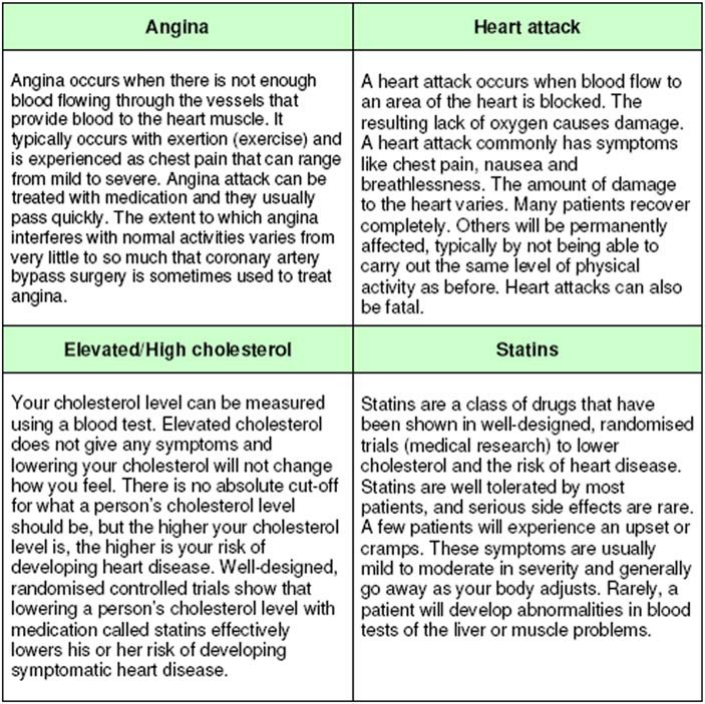
Textual information presented to participants

We conducted an Internet-based randomized trial comparing six summary statistics to express risk reduction (Figure 1). We wanted to conduct Internet-based studies because we assumed that this would be an efficient way to recruit participants and conduct trials of different presentations. We first presented information about the study and asked participants to give informed consent to participate. We then asked them to imagine that they had elevated cholesterol and needed to decide whether or not they would start taking statin therapy. We presented textual information to the participants about elevated cholesterol and the increased risk of developing coronary heart disease (CHD), i.e. angina or having a heart attack, during the next ten years; about the need to take a statin pill each day and the side-effects of taking statins (Figure 2); and that the estimated out-of-pocket cost for statin treatment was US $50 per month.

### Elicitation of values

**Figure 3:**
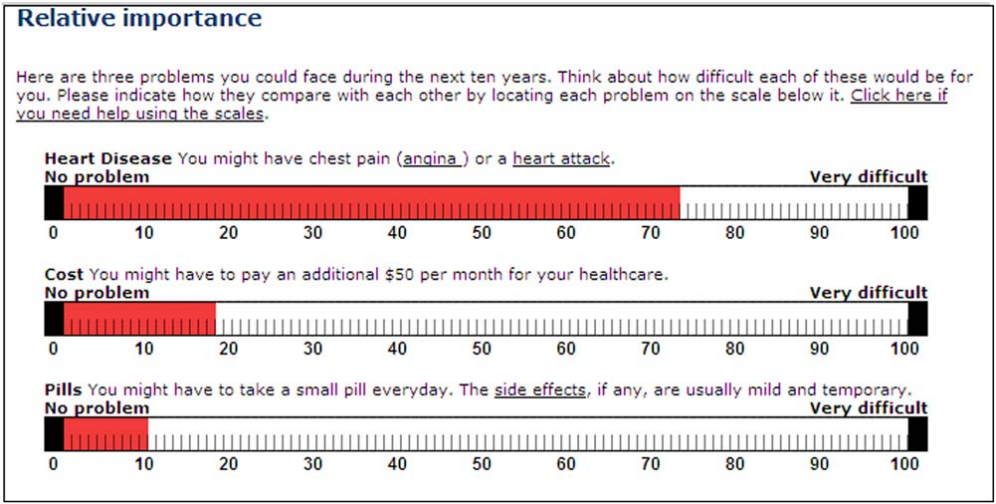
Visual analogue scales used to elicit participants' values

**Figure 4:**
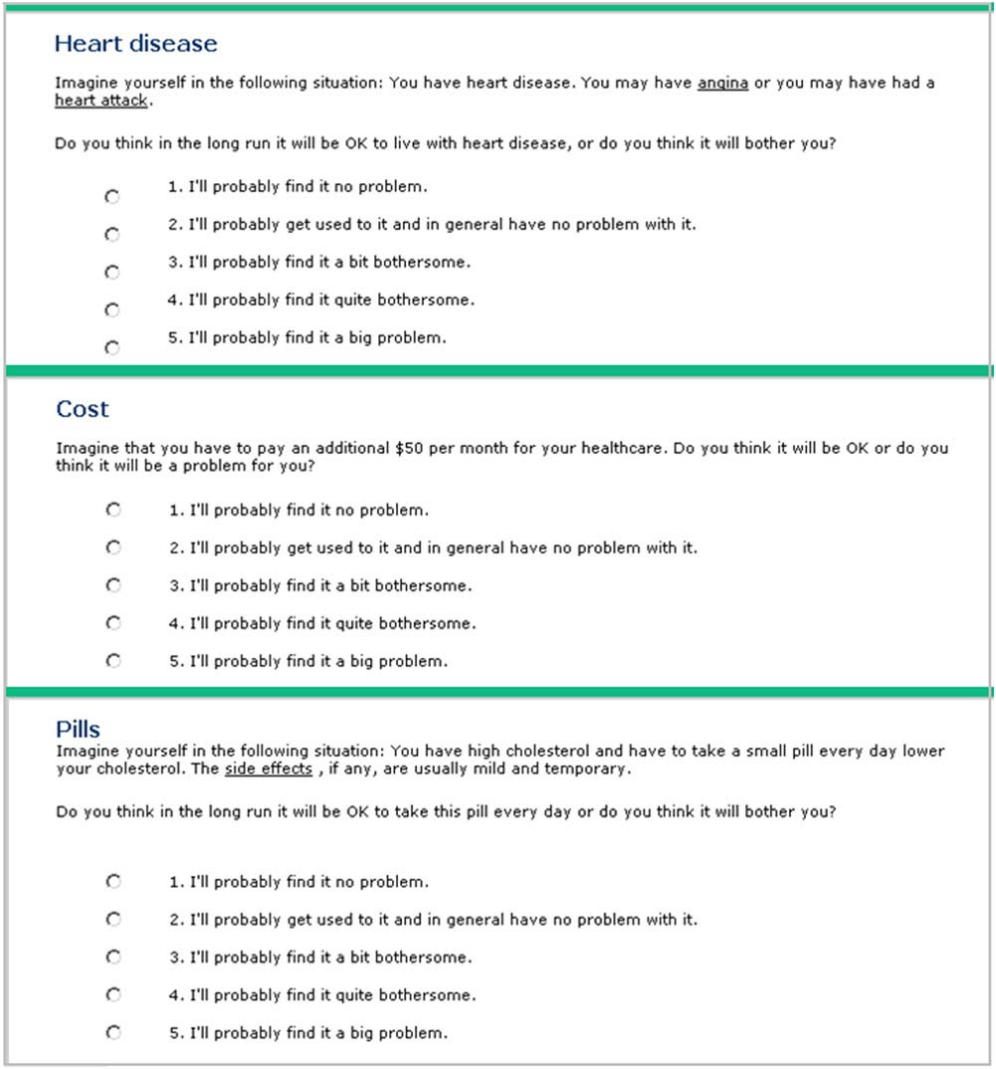
Category rating scales used to elicit participants' values

We chose to compare two methods of eliciting values, the category rating scale (CRS) with five response options and the visual analogue scale (VAS), range 0–100, that were simple to administer on the Internet without participant training. We elicited participants' values for three consequences of the choice to take statins (CHD, out-of-pocket cost, and taking a pill every day) using both VAS (Figure 3) and CRS (Figure 4). We randomized the participants to the order of administration of these two methods.

### Presentations

We then randomised participants a second time to view one of six summary statistics expressing the reduced risk of CHD with statin therapy (Box S1). We chose four summary statistics based on the results of systematic reviews of previous studies [6], including our own (unpublished data available from the authors): the RRR, ARR, NNT and event rates (ER). These earlier studies showed that individuals perceived the same effects to be greater when stated as the RRR compared to the ARR. Studies comparing the RRR and NNT found the RRR to be significantly more persuasive. In studies comparing the ARR and NNT, there was inconclusive evidence as to persuasiveness. In studies to find the minimally important difference, the ARR produced 20% larger differences in the medians than the NNT (25% versus 5%). Also, the RRR was found to be more persuasive than ARR, NNT, and percent event-free patients. In addition to these four summary statistics, we presented “Tablets needed to take” (TNT) proposed by Skolbekken [33] and the whole numbers presentation (WN) proposed by Hollnagel [34] (natural frequencies) (Box S1). Of the six summary statistics, RRR is a relative measure and the other five are absolute measures of effect.

For our risk reduction presentation, we assumed a 10-year baseline risk for CHD of 6% without statins [35], which is the estimated risk for a person without other risk factors than a high cholesterol level, and an RRR for CHD with statin therapy of 30 % [36]. We calculated the other summary statistics based on these two values. Participants were given information, using their allocated summary statistic, about the reduced risk of CHD with statin therapy and then asked to indicate if they would decide to start taking statins. The only allowed choices were “yes” or “no”. Participants could access explanations of heart disease, statins and side effects using hyperlinks (Figure 2). They were not provided any additional explanation of the summary statistics that they were shown (e.g. RRR or ARR) (as shown in the Box S1).

### Recruitment, eligibility and allocation

We contracted a vendor to send emails to 700,000 consumers in the US who had “opted-in” to receive messages concerning health and physical fitness. Participants were offered the option of participating in a lottery to receive a $100 gift certificate as an incentive to participate. Only participants who identified themselves as at least 18 years old and as literate in English were included in the analyses. Allocation to the order in which the two value-elicitation methods were administered was block-randomized. Allocation to one of the six summary statistics was also block-randomised, using a looped sequence of 600 presentation assignments consisting of 100 blocks of six that was generated on http://www.randomization.com.

### Data collection

We collected demographic data, including sex, age, years of education, country of residence and profession after the participants decided whether they would start taking statins. In addition, as described in Appendix S1 and Appendix S2, we asked two questions to assess their numeracy and three questions about their experience with CHD and hypercholesterolemia to assess the salience of the scenario (i.e. how relevant or important the hypothetical scenario was likely to be to the participants). We then asked them questions about their decision, including their level of confidence in their decision (on a 5 point scale from ‘Not at all confident’ to ‘Extremely confident’) and about themselves. Finally, we showed them all six summary statistics and asked which one they preferred.

Participants' responses to the questions on the HIPPO website were entered directly into a database where the data were stored anonymously. Confidentiality of the data was ensured by not collecting information that would make it possible to identify the participants. Voluntary contact information that participants supplied in order to request a report of the study results or to participate in the lottery was stored in a separate database so it was not possible to couple contact information and responses.

### Statistical analyses

We assessed the relative merits of using VAS and CRS to elicit values by comparing their distributions, response rates, and expected utilities expressed as relative importance scores (RIS), as described in Appendix S1. Spearman rank correlation coefficients and box-plots were made for the elicited VAS and CRS scores. To compare user acceptability, we used a Chi-squared test to compare the 100% response rate for VAS (i.e. completion of all 3 questions) when it was administered first and the 100% response rate for CRS when it was administered first.

The analysis of the concordance of participants' elicited values and their decisions was first performed using the elicited VAS-values. The three scales (CHD, cost and pills) were combined using four approaches to weighting them to derive relative importance scores for each participant. 1) We subtracted a rough estimate of the expected utility (EU) of taking statins from the expected utility of not taking statins, using the individual's response to the VAS for CHD, cost and pills and the probability of each of these consequences to calculate RIS_EU_VAS_. 2) We used principal component analysis (PCA) to derive the weights used to calculate RIS_PCA_VAS_. 3) We used logistic regression (LR) to derive the weights used to calculate RIS_LR_VAS_. 4) We used equal weights (ONE) to calculate RIS_ONE_VAS_. In weighting schemes 2, 3 and 4, the relative importance of the undesirable consequences (Pills and Cost) was subtracted from the relative importance of the desirable consequences (reduced risk of CHD). The weights are presented in Table 1 and the formulae used to calculate them are described in Appendix S1. The CRS-values were combined for the three scales using the same four approaches to derive RIS values for each participant.

**Table 1:** Weighting used to derive the relative importance scores (RIS)

	Weights		
Relative importance Scores*	CHD	Pills	Cost
RIS_EU_			
_ _VAS	0.04/0.06	1/0	1/0
CRS	0.04/0.06	1/0	1/0
RIS_PCA_			
VAS	0.004	−0.588	−0.809
CRS	0.044	−0.514	−0.856
RIS_LR_			
_ _VAS	0.011	−0.013	−0.005
CRS	0.125	−0.476	−0.162
RIS_ONE_			
_ _VAS	1	−1	−1
CRS	1	−1	−1

* VAS = visual analogue scale; CRS = category rating scale; RIS = relative importance score. RIS_EU_ weights were based on an approximation of participants' expected utility (EU) for a decision (the expected utility of taking statins minus the expected utility of not taking statins using the probabilities shown in the table).

RIS_PCA_ weights were based on principle component analysis (PCA).

RIS_LR_ weights were based on logistic regression (LR).

RIS_ONE_ weights were equal weights (ONE).

For RIS_EU_, the estimate of the expected utility (EU) of taking statins was subtracted from the estimate of the expected utility of not taking statins.

For RIS_PCA_, RIS_LR_, and RIS_ONE_ the undesirable consequences (Pills and Cost) were subtracted from the desirable consequences (reduced risk of CHD).

The formulae used to calculate the weights used for each of the four approaches to weighting (RIS_EU_, RIS_PCA_, RIS_LR_, and RIS_ONE_) can be found in Appendix S1.

In order to compare the effects of the different summary statistics on decisions in relation to elicited values, we performed logistic regression analyses for each of the six groups and for the pooled group of absolute summary statistics (i.e. all summary statistics except for RRR). The participant's decision was the dependent variable and RIS was the predictor. We compared the intercepts and slopes of the logistic regressions for each of the six summary statistics and for the pooled absolute summary statistics. We compared the likelihood of participants deciding to take statins (expressed as log odds) across the six presentation groups at three values of RIS in order to examine the impacts of the different presentations for people with a range of values. The three values were the points at which the regression line for all five of the groups shown one of the absolute summary statistics crossed log odds = 0 (odds = 1; i.e. where there was a 50% likelihood of their deciding to take statins), and the 1^st^ and 3^rd^ quartiles of RIS.

We compared the four models using VAS and the four models using CRS and used the c-statistic (a measure of concordance), which is equal to the area under the receiver-operating characteristic (ROC) curve when the outcome is binary, to compare the discriminatory ability of the logistic regressions fitted for all RIS models for each summary statistic, i.e. 48 c-estimates [37]. A c-statistic of 1.0 indicates perfect accuracy, while a c-statistic of 0.5 indicates a non-discriminatory test.

We explored the relationship between the decision of whether to start taking statins in a logistic regression using RIS_ONE_VAS_ and presentation group as explanatory variables and the following covariates: numeracy, salience, sex, professional background, education and age.

Finally, we summarized the participants' level of confidence and satisfaction in their decisions; and the number and percent of participants who preferred each of the six summary statistics, which they indicated after they had seen all six.

We had no prior information as a basis for calculating a sample size for this pilot study. The number of participants in each group in the pilot study was therefore based on power calculations for detecting a medium and a somewhat larger effect size for the correlation between the VAS and CRS scores, as suggested by Cohen [38]. Based on an effect size index (q) of 0.30 to 0.40, an alpha of 0.05 to 0.10, and power of 0.70 to 0.80, we estimated that we would need between approximately 80 and 140 participants per group. No corrections for multiple testing were performed for the tests reported here. The p-values should be interpreted with caution and regarded as hypothesis generating.

## Results

### Feasibility

Five weeks after emails were sent to approximately 700,000 people, there were 1,492 log-ons to the study site, resulting in 782 complete records between 31 October and 4 December 2002. Of these, one was excluded because age was less than 18. Eleven other records with a VAS score for CHD of zero were excluded because we assumed that the participants had either misunderstood the question or had not provided a serious response. We manually checked whether participants completed the study more than once. As we found no evidence for that, the remaining 770 records were included in the analyses. The distribution of age, sex, country of residence, years of education, profession, numeracy, and salience score among the six presentation groups shows that the randomization process worked well, providing comparable groups (Table 2). Fifty-eight percent of the participants were women, 62% were between 40 and 59 years old, 47 % had 17 or more years of education and another 43% had 13 to 16 years of education, 84% were from the U.S.A., 23% were health professionals and 17% were scientists or engineers.

**Table 2:** Participant characteristics

	RRR n (%)	ARR n (%)	NNT n (%)	ER n (%)	TNT n (%)	WN n (%)	Total n (%)
n	131	120	131	135	121	132	770
**Women**	75 (57)	68 (57)	72 (55)	81 (60)	75 (62)	77 (58)	448 (58)
**Age**							
18–29	7 (5)	10 (8)	6 (5)	10 (7)	8 (7)	10 (8)	51 (7)
30–39	16 (12)	11 (9)	15 (11)	16 (12)	17 (14)	16 (12)	91 (12)
40–49	35 (27)	33 (28)	32 (24)	43 (32)	36 (30)	33 (25)	212 (28)
50–59	45 (34)	44 (37)	46 (35)	40 (30)	40 (33)	49 (37)	264 (34)
60–69	21 (16)	13 (11)	20 (15)	19 (14)	14 (12)	23 (17)	110 (14)
70–79	7 (5)	4 (3)	11 (8)	6 (4)	4 (3)	0	32 (4)
over 80	0	5 (4)	1 (1)	1 (1)	2 (2)	1 (1)	10 (1)
**Years of education**							
8 years or less	1 (1)	0	0	0	1 (1)	3 (2)	5 (1)
9–12 years	15 (11)	12 (10)	14 (11)	10 (7)	12 (10)	10 (8)	73 (9)
13–16 years	56 (43)	45 (38)	54 (41)	67 (50)	49 (40)	59 (45)	330 (43)
17 years or more	59 (45)	63 (53)	63 (48)	58 (43)	59 (49)	60 (45)	362 (47)
**Country of residence**							
Canada	4 (3)	9 (8)	6 (5)	7 (5)	4 (3)	8 (6)	38 (5)
Germany	1 (1)	0	0	0	0	1 (1)	2 (0)
Norway	0	0	0	0	1 (1)	2 (2)	3 (0)
USA	110 (84)	97 (81)	113 (86)	109 (81)	104 (86)	111 (84)	644 (84)
other	16 (12)	14 (12)	12 (9)	19 (14)	12 (10)	10 (8)	83 (11)
**Profession**							
GP or Health professional	34 (26)	29 (24)	31 (24)	27 (20)	23 (19)	33 (25)	177 (23)
Scientist or engineer	22 (17)	23 (19)	20 (15)	25 (19)	18 (15)	22 (17)	130 (17)
**Numeracy**							
0 (low)	16 (12)	17 (14)	15 (11)	14 (10)	12 (10)	11 (8)	85 (11)
1	50 (38)	33 (28)	47 (36)	31 (23)	35 (29)	36 (27)	232 (30)
2 (high)	65 (50)	70 (58)	69 (53)	90 (67)	74 (61)	85 (64)	453 (59)
**Salience**							
0	3 (2)	5 (4)	4 (3)	6 (4)	9 (7)	8 (6)	35 (5)
1	58 (44)	59 (49)	59 (45)	69 (51)	65 (54)	61 (46)	371 (48)
2	42 (32)	35 (29)	44 (34)	45 (33)	31 (26)	41 (31)	238 (31)
3	10 (8)	4 (3)	8 (6)	5 (4)	4 (3)	2 (2)	33 (4)
4	18 (14)	17 (14)	16 (12)	10 (7)	12 (10)	20 (15)	93 (12)

### Elicitation of values

**Figure 5:**
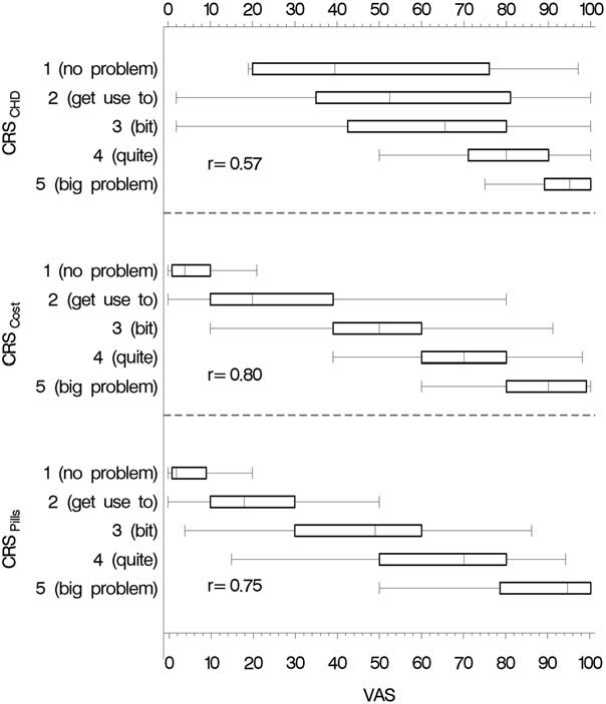
Category rating scale (CRS) elicited values mapped on visual analogue scale (VAS) elicited values

**Figure 6:**
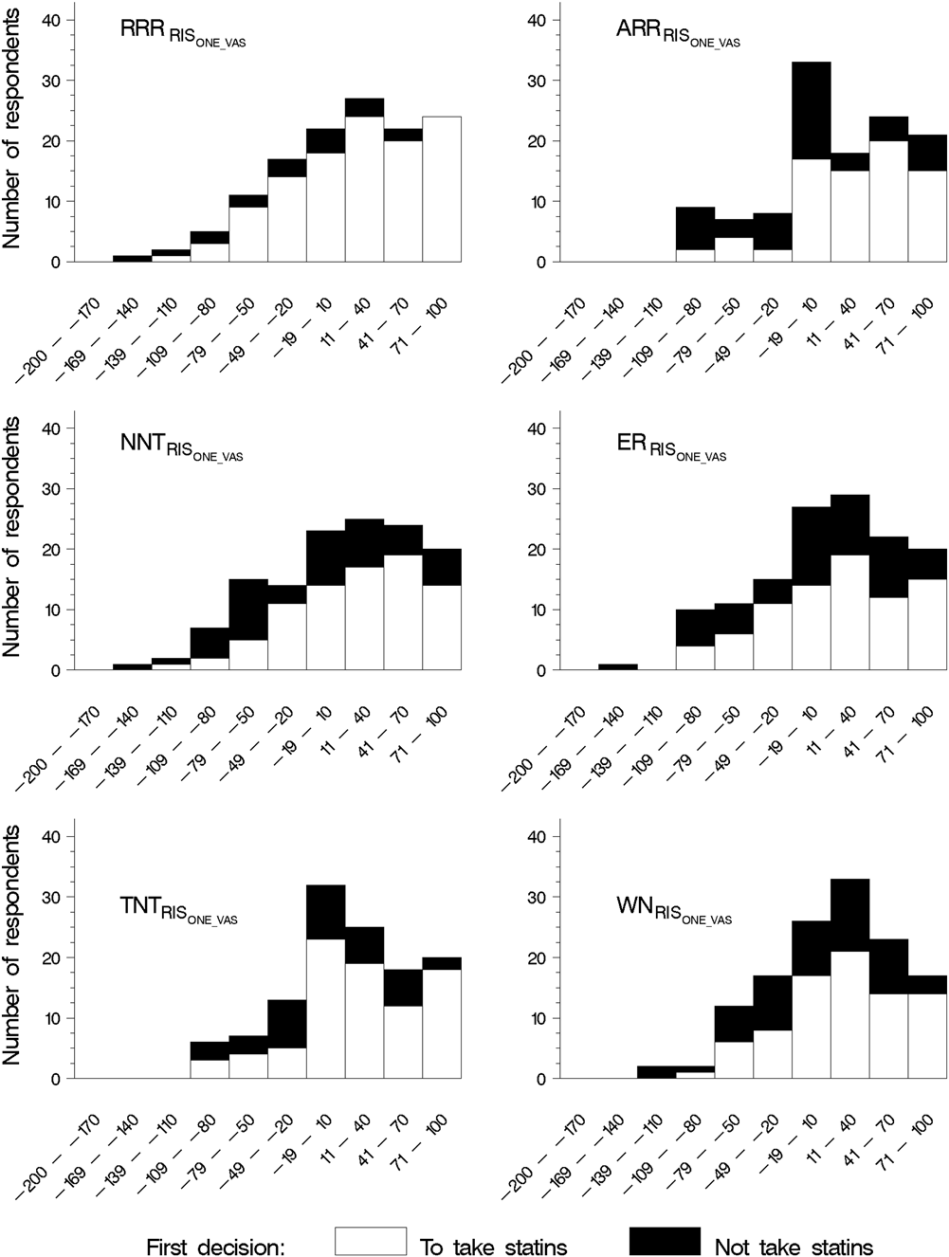
Distribution of RIS scores derived from VAS values

Of the 1492 log-ons, 998 people (67%) went as far as the first value elicitation exercise, with 509 (51%) in the VAS-first group and 489 (49%) in the CRS-first group. In all, 443 (87%) of the VAS-first group completed all three visual analogue scales, while 446 (91%) of the CRS-first group completed all three category rating scales (p = 0.03). VAS and CRS correlated well for cost (r = 0.80) and pills (r = 0.75). For CHD, the correlation was lower (r = 0.57). The median VAS scores for the five CRS categories for CHD, cost and pills were approximately equidistant (Figure 5). There was no difference in the distribution of the elicited raw value scores (VAS and CRS) nor the RIS between the summary statistics presentation groups (Figure 6).

**Figure 7:**
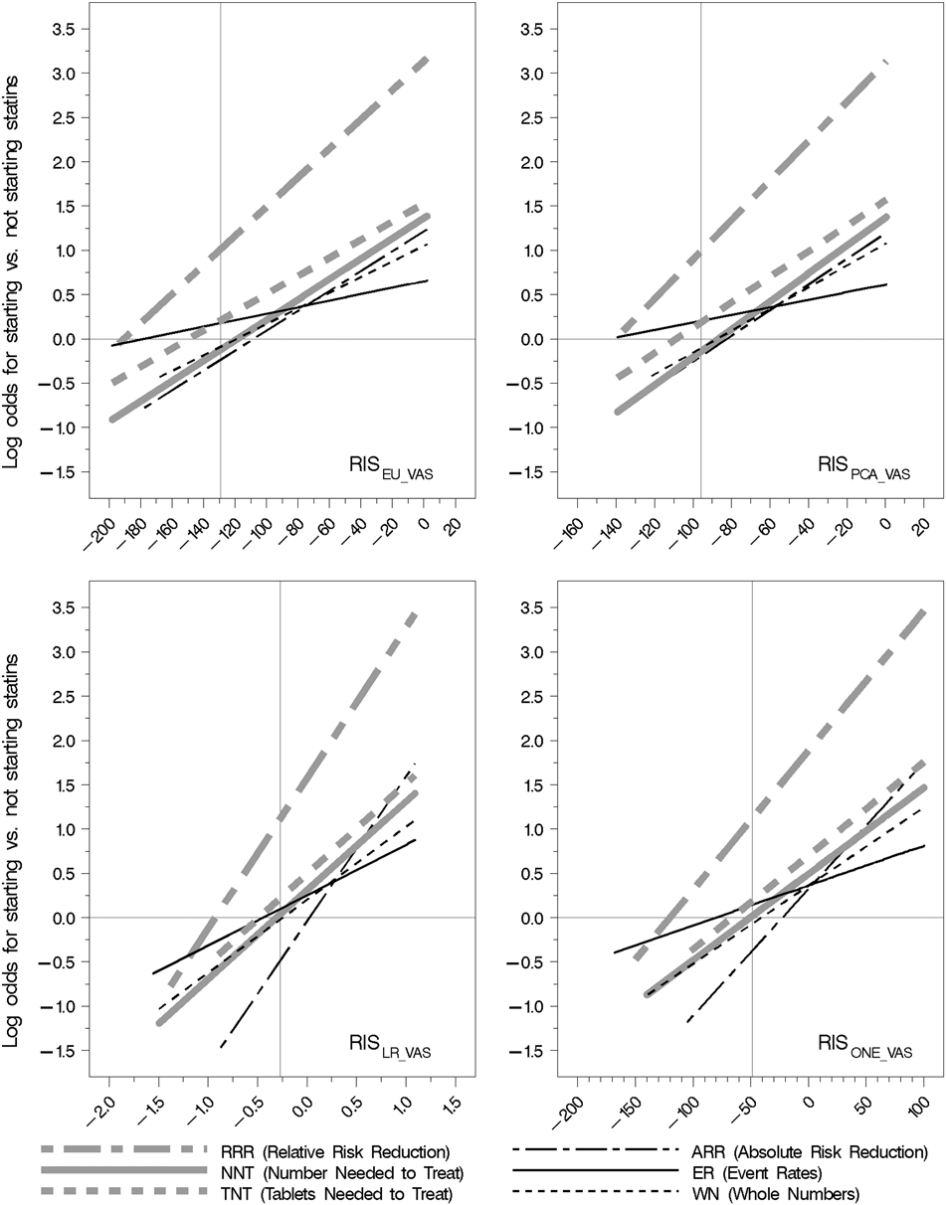
Log odds for deciding to start taking statins in relation to relative importance scores

From a visual inspection of the linear predictors produced by regressing participants' decisions on their relative importance score (RIS) derived from VAS values (RIS_VAS_) and on RIS derived from CRS values (RIS_CRS_), it appeared that there were no important differences between them that would indicate that either VAS or CRS was superior. Neither did it appear that any one of the RIS models (RIS_EU_, RIS_PCA_, RIS_LR_, RIS_ONE_), derived using the weights in Table 1, was better than the others at discriminating between “yes” and “no” decisions (Table 3 and Figure 7).

**Table 3:** C-values for logistic regressions: Four models of calculating RIS for visual analogue scales (VAS) scores and categorical rating scale (CRS) scores*

	RRR	ARR	NNT	ER	TNT	WN	Overall
**Relative importance scores (RIS) based on VAS, c-values**							
RIS_EU_	0.731	0.650	0.655	0.543	0.646	0.622	0.649
RIS_PCA_	0.720	0.636	0.650	0.535	0.639	0.616	0.645
RIS_LR_	0.751	0.709	0.652	0.576	0.627	0.611	0.656
RIS_ONE_	0.728	0.697	0.649	0.553	0.643	0.617	0.657
**Relative importance scores (RIS) based on CRS, c-values**							
RIS_EU_	0.794	0.647	0.694	0.598	0.659	0.629	0.667
RIS_PCA_	0.772	0.635	0.685	0.582	0.662	0.621	0.660
RIS_LR_	0.832	0.657	0.682	0.614	0.630	0.653	0.671
RIS_ONE_	0.769	0.665	0.666	0.587	0.642	0.630	0.660

* A c-statistic of 1.0 indicates perfect accuracy, while a c-statistic of 0.5 indicates a non-discriminatory test. The weights used in the four models are shown in Table 1.

### Decisions and responses

Altogether, 67% of the participants said they would start taking statins. There was a statistically significant difference in the percent of participants that decided to start taking statins across the six groups, with the RRR group having the highest proportion (86%) compared to the others (range 60% to 69%, p<0.0001) (Table 4).

**Table 4:** Decision to start taking statins and other responses

	RRR n (%)	ARR n (%)	NNT n (%)	ER n (%)	TNT n (%)	WN n (%)	Total n (%)
n	131	120	131	135	121	132	770
**Decision to take statins (yes)**	113 (86)	75 (63)	83 (63)	81 (60)	84 (69)	81 (61)	517 (67)
**Preferred presentation**							
Not stated	0	(1)	(1)	(2)	(3)	(1)	8 (1)
RRR	(59)	(56)	(49)	(53)	(40)	(48)	393 (51)
ARR	(4)	(3)	(2)	(4)	(3)	(5)	29 (4)
NNT	(3)	(4)	(6)	(4)	(4)	(5)	34 (4)
ER	(12)	(12)	(10)	(19)	(16)	(13)	104 14)
TNT	0	(2)	(4)	0	(2)	(1)	11 (1)
WN	(22)	(22)	(28)	(18)	(32)	(27)	191 (25)
**Sure of rated relative importance**							
1 (low)	0	1 (1)	0	1 (1)	0	0	2 (0)
2	7 (5)	4 (3)	4 (3)	7 (5)	8 (7)	6 (5)	36 (5)
3	15 (11)	16 (13)	14 (11)	13 (10)	18 (15)	14 (11)	90 (12)
4	44 (34)	51 (43)	44 (34)	53 (39)	38 (31)	54 (41)	284 (36)
5 (high)	65 (50)	48 (40)	69 (53)	61 (45)	57 (47)	58 (44)	358 (47)
**Confidence in decision**							
Low confidence	30 (23)	31 (26)	26 (20)	35 (26)	30 (25)	37 (28)	189 (25)
High confidence	101 (77)	89 (74)	105 (80)	100 (74)	91 (75)	95 (72)	581 (75)
**Understanding of information**							
1 (low)	0	0	0	0	2 (2)	0	2 (0)
2	4 (3)	8 (7)	4 (3)	6 (4)	4 (3)	3 (2)	29 (4)
3	15 (11)	9 (8)	16 (12)	14 (10)	10 (8)	9 (7)	73 (9)
4	28 (21)	33 (28)	25 (19)	38 (28)	34 (28)	33 (25)	191 (25)
5 (high)	84 (64)	70 (58)	86 (66)	77 (57)	71 (59)	87 (66)	475 (62)

There were no statistically significant differences across groups regarding which summary statistic they preferred or in their confidence in decisions (Table 4). However, of the 762 participants who indicated their preferred summary statistic after viewing all six, 393 (52%) preferred RRR, compared to the others (range 1% to 25%, p = 0.07) (Table 4).

The log odds for the four groups other than event rates (ER) and RRR were similar at all values of the relative important scores (RIS). The log odds for the RRR group was significantly (p = 0.0007) greater at all values of RIS (Figure 7), indicating that the proportion of people deciding to take statins was larger than for the other five presentations, independent of participants' values. The RRR and the ARR groups had the steepest slopes (β = 0,016, 95% CI 0.006 to 0.025, and β = 0,014, 95% CI 0.006 to 0.022, respectively). The ER group had the flattest slope (β = 0,005, 95% CI-0.002 to 0.011) and was the only group that had a regression line that was not significantly different from zero.

For the pooled group of absolute summary statistics, the value of RIS_ONE_VAS_ was −48.5 at log odds for starting statins = 0 (odds = 1). At this value of RIS, the odds for the RRR group was three times the odds for the other five groups (log odds 1.124, odds 3.1). At the 1^st^ and 3^rd^ quartiles of RIS_ONE_VAS_ (−20 and 51) the odds for RRR was respectively 3.7 and 5.8 times that of the absolute summary statistics.

### Explanatory factors

Sex (p = 0.51) and age (p = 0.40) were not statistically significant explanatory factors for the decision to take pills. Nor was there a significant difference between the proportion of all health professionals or general practitioners (68%) and others (67%) who decided to start taking statins (p = 0.98). Scientists and engineers, on the other hand, were less likely to decide to start taking statins (56%) than both general practitioners and the rest of the study population (69%, p = 0.003). Participants with the highest numeracy score (2) also decided to start taking statins (62%) less often than those with a numeracy score of one (73%) or zero (75%) (p = 0.004). Similarly, participants with 17 or more years of education were less likely to take satins (62%) compared to those with 13–16 years of education (72%) and those with 12 or less years of education (71%) (p = 0.032).

We estimated the saliency of the scenario for participants based on questions about whether participants had CHD, knew their cholesterol level, and knew anyone who had experienced CHD (see S1). Based on a summary of their responses to these three questions, the more salient the scenario was likely to be to participants (score 0 to 4), the more likely the participants were to decide to take statins (p = 0.01). Among those with high salience scores (3 or 4) 76% would start taking statins compared to 71%, 63% and 54% for those with lower salience scores of two, one, and zero respectively.

## Discussion

The proportion of participants who chose to take statins was highest for the RRR group. This was expected, as had been shown in previous trials (and since confirmed in subsequent trials), that presenting the RRR is more likely to result in decisions to recommend or accept an intervention than the ARR or NNT [6]–[12]. The RRR and ARR groups had the steepest slopes (Figure 4) and the ER group had the flattest slope and the only one that was not significantly different from zero, suggesting that decisions made in this group were independent of the participants' RIS values.

Based on these observations, we generated the following hypotheses regarding the concordance between decisions and values to be tested in a confirmatory study using the methods developed in this pilot:

1. RRR results in a higher likelihood of deciding to start taking statins across RIS values compared to the absolute summary statistics.
2. The slope of the log odds of ARR is greater than the slope of the other absolute summary statistics.
3. The concordance between decisions and values for ER is less than for the other absolute summary statistics; i.e. that the slope for the relationship between RIS values and the log odds of deciding to take statins is not significantly different from zero for ER (indicating that decisions were independent of the participants' elicited values), whereas it is positive (consistent with what would be predicted) and significantly different from zero for the other absolute summary statistics.

We estimated that we would need about 750 to 800 subjects in each group to test these hypotheses based on the results of our pilot study.

### Feasibility

We found that the biggest challenge to this Internet-based trial was recruiting a sufficient number of participants to achieve adequate sample size, similar to what has been found for surveys [39] and in a study similar to ours [40]. Only about 52% of log-ons to our website resulted in complete, usable records compared to 72% in the latter study. The relative success of that study may be attributable to intensive recruiting efforts on websites and in printed materials dedicated to patients with the disease used in the scenario and their carers.

A related problem with conducting this type of study on the Internet is uncertainty about the applicability of the findings, as discussed below. In this study we contracted for 700,000 e-mail invitations to be sent out but we do not have data to compare the characteristics of participants to those who were invited to participate. Nor do we know how many invitations actually reached their addressees or how many additional people participated who were not among those to whom the invitations were sent.

### Elicitation and weighting of values

We elicited participants' values for three consequences that we thought would be most important to people making a decision in this scenario. We did not attempt to identify other concerns that individual participants may have had, and it is possible that they might have taken other elements into consideration in making their decisions. However, on average the likelihood that participants would decide to start taking statins was correlated with the relative importance of these three consequences, as predicted.

In measuring subjective change in pulmonary function, Guyatt and colleagues found a seven-point category rating scale (CRS) somewhat easier to use than the visual analogue score (VAS) and responsiveness was comparable [41]. Intuitive grasp of the minimal important difference guides the choice of how many points to have on a scale for this purpose. Badia and colleagues [42] found direct correspondence between participants' ratings of their overall health on a 5-point CRS and VAS, although the CRS values were unevenly distributed along the VAS; and Schünemann and colleagues found direct correspondence on 7-point health related quality of life instruments [43].

The fact that we found correlation between VAS and category rating scales (CRS) is not sufficient to justify the use of either one of them. Using a 5-point CRS, it is difficult to interpret the results when using three explanatory variables (CHD, pills, cost) as there would be 125 different groups. Because there would be too few observations for many of the groups, reliability of the resulting log odds ratios could not be assumed. A solution to this is to treat the CRS values as continuous variables. However, certain assumptions must be fulfilled. It appears that the CRS fulfils the assumption that the categories are ordered and the condition that they are equidistant, if one uses their placement on the VAS-scale as evidence of the subjective values of the categories. This does not correspond with Badia's findings [42] of uneven distribution. However, we did find a clustering of the categories at the higher end of the VAS, as reported by Badia. In addition, because we found a clustering of individuals' VAS around 10, 20, etc., we will remove these labels from the VAS in future studies, leaving only the low and high anchor points of “0” and “100” respectively.

The profiles of the estimates of the relative importance scores based on the VAS and the CRS were similar. Being able to use a continuous variable in the logistic regressions, instead of an approximation using a categorical variable, outweighs the slightly higher response rate of the CRS (4%), so we have decided to use VAS in future studies.

As illustrated in Figure 4, there was little difference across the four ways we used to derive the relative importance scores (RIS) using the weights shown in Table 1. The C-values in Table 3 show that any weighting method yields a model that discriminates between a “yes” and “no” decision to start taking statins about as well as any other, consistent with Dawes' findings that “improper” linear models that use equal weighting are quite robust for making clinical predictions [44]. Guided by the principle of parsimony, we chose the simplest model (RIS_ONE_VAS_) for the subsequent HIPPO studies, i.e. equal weights. The absolute RIS values are arbitrary and cannot be compared across studies using different scenarios. However, the results of this study suggest that the RIS scores provide a robust measure of the relative importance that participants attach to the consequences of a decision for comparisons within a study, regardless of the weights that are used.

### Explanatory factors and applicability of the results

Participants with a scientific background, who were more numerate, or who had more years of education were less likely to decide to start taking statins. General practitioners and the general public had the same likelihood to start taking statins, in contrast to participants who classified themselves as scientists, who were less likely to opt for statin therapy. The likelihood of deciding to start statins also increased as the salience of the scenario increased. This finding could be explained by the availability heuristic [45], which suggests that as vividness or emotional impact increases (in this case the salience of the scenario), the perceived probability of an outcome increases (in this case CHD).

These findings suggest that the effects of different presentations of risk may interact with these characteristics and that the applicability of the results of trials such as this one might be limited in relationship to these characteristics. Furthermore, it is uncertain to what extent results from hypothetical scenarios apply to actual decisions [7], [46]. While the results of Internet-based studies such as this one likely apply to printed information as well as electronic information, the relevance of the results to personal communication is uncertain.

The applicability of the results to different populations is also uncertain, particularly to less educated populations. Most (86%) of the participants were from the U.S.A. and 47% had 17 years or more of education. By comparison, only 8% of the U.S. population had a master's degree or higher (roughly comparable to 17 years or more of education) in 2002 (http://www.census.gov/population/socdemo/education/ppl-169/tab11.xls). In light of the finding that highly educated participants appeared less likely than others to decide to start taking statins across presentations, it is possible that they would also respond differently to different presentations, thereby limiting the applicability of findings from Internet-based studies, such as this, to populations with less education. Similarly, the applicability of the results to populations for whom the scenario is more or less salient may be limited.

A systematic review of the impact of different presentations on treatment decisions by patients found that, although good quality studies were limited in number, the results suggested that framing effects were influenced by various effect modifiers [10]. Malenka and colleagues [47] found that those with higher education or being treated for the condition were more likely to prefer medication when presented the RRR, and Misselbrook and colleagues [48] found that those with hypertension or taking other chronic medications (which could be considered as indicators of saliency) were more likely to accept treatment when presented the RRR, although there was not a significant difference in responses in relationship to familiarity with stroke. Other studies that examined education as a possible effect modifier for framing effects did not find a significant effect [49], [50].

### Conclusions

It is feasible to conduct randomized trials of different ways of presenting the effects of health care on the Internet. However, recruitment of participants is a major challenge. In addition, although randomisation ensures comparable groups, questions may still remain about the applicability of the results to specific populations. Visual analogue scales appear to function well for eliciting the relative importance of the consequences of a decision.

Our approach to comparing different ways of presenting information about the effects of health care is, so far as we are aware, the first attempt to evaluate the extent to which different presentations help people to make decisions that are consistent with their own values. The validity of our approach is supported by the fact that the likelihood of participants deciding to start taking statins increased as predicted in relationship to the relative importance they placed on the advantages and disadvantages of taking statins; and by the consistency of our results with what could be hypothesised based on previous studies, i.e. that participants who were shown the relative risk reduction were more likely to decide to take statins regardless of their values compared with participants who were shown any of the five absolute summary statistics.

## Supporting Information

Box S1 The six presentations of risk
(0.06 MB TIF)

Appendix S1 Methods
(0.04 MB DOC)

Appendix S2 Numeracy and salience
(0.06 MB TIF)

Checklist S1 (0.05 MB DOC)

Protocol S1 HIPPO 1. What is the effect of the summary statistic used to present the benefits of statins on decisions about whether to use them?
(0.13 MB DOC)

Protocol S2 Facsimile of HIPPO 1 webpages
(0.88 MB PPT)
