# Supplementary figures and images for: How Should the Impact of Different Presentations of Treatment Effects on Patient Choice Be Evaluated? A Pilot Randomized Trial
Source: PLoS One. 2008 Nov 24;3(11):e3693. doi: 10.1371/journal.pone.0003693 (PMC2585274; doi:10.1371/journal.pone.0003693)

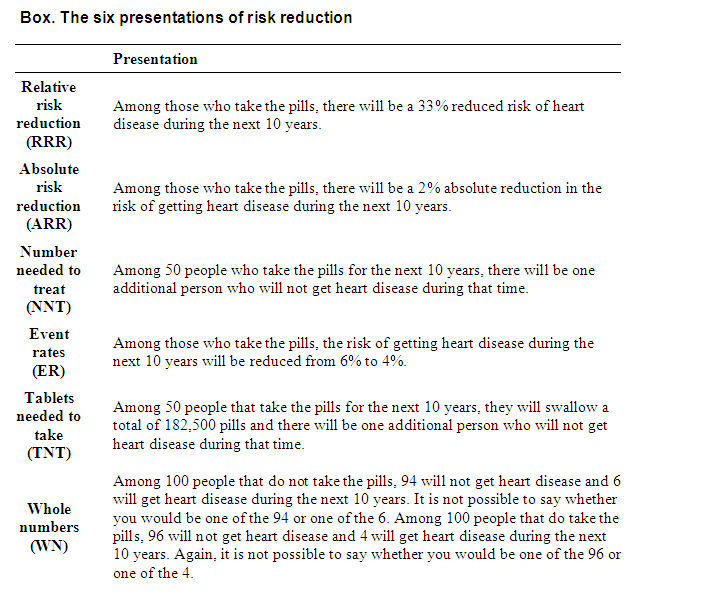

Supplement: Box S1 — The six presentations of risk (0.06 MB TIF) [file pone.0003693.s001.tif]

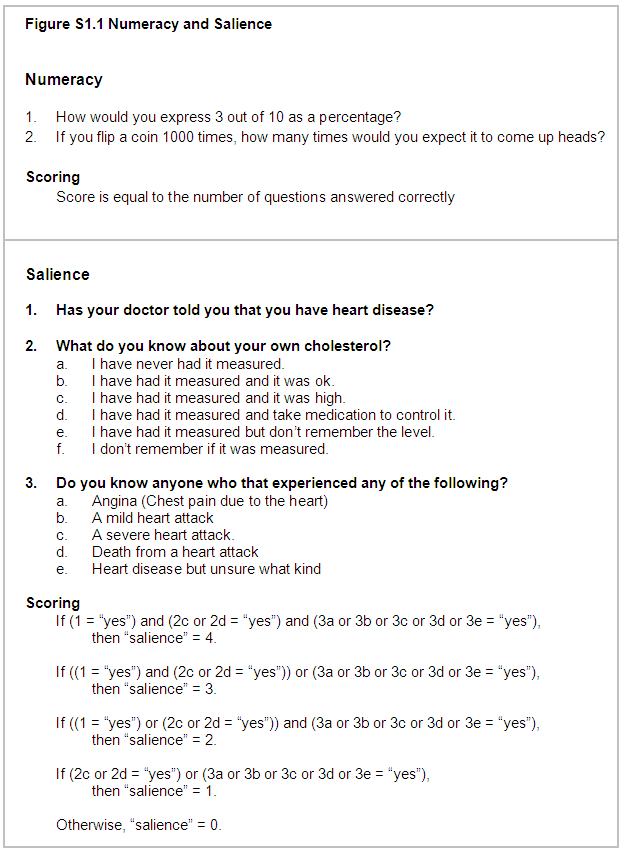

Supplement: Appendix S2 — Numeracy and salience (0.06 MB TIF) [file pone.0003693.s003.tif]

## Slide 1
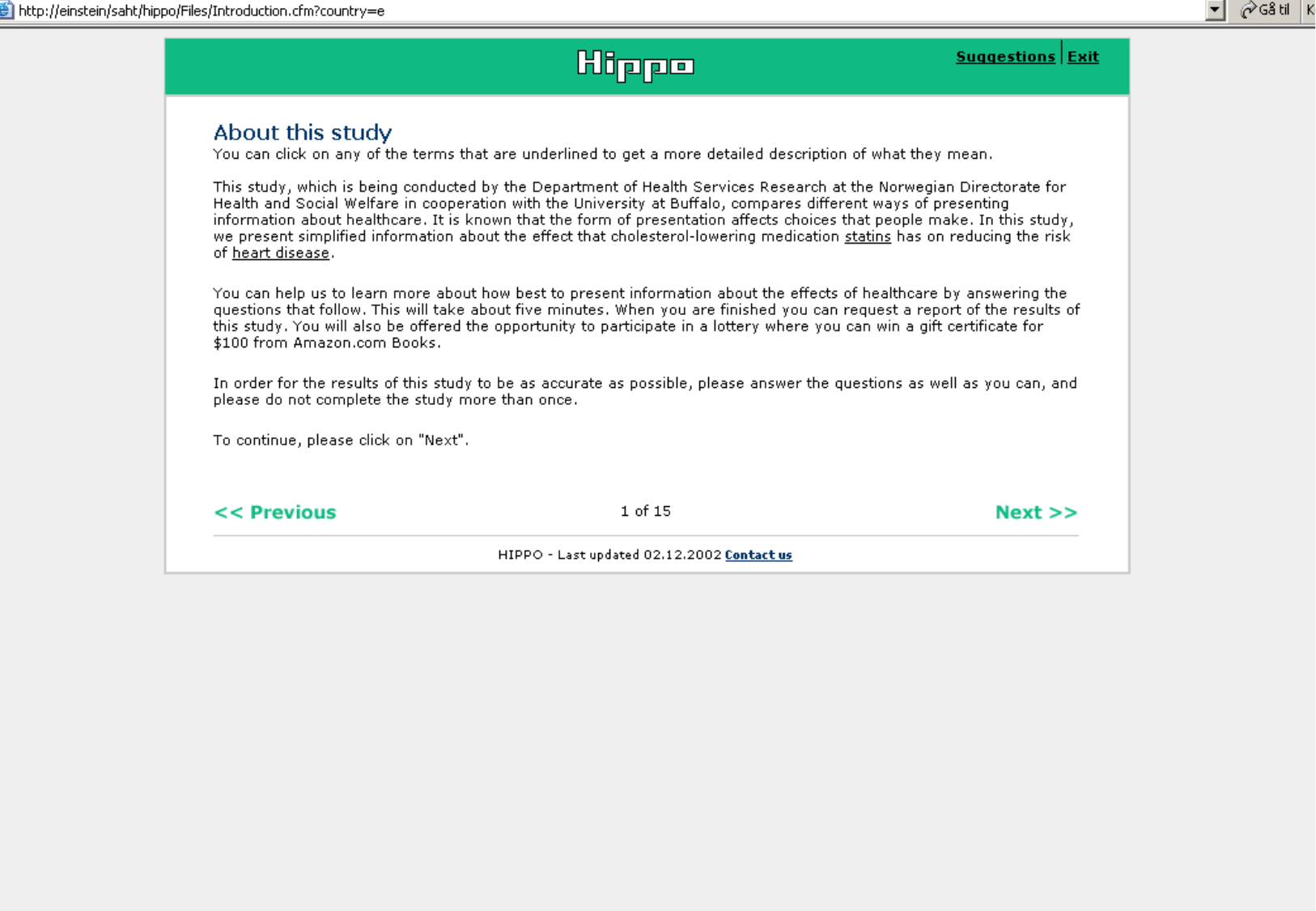

## Slide 2
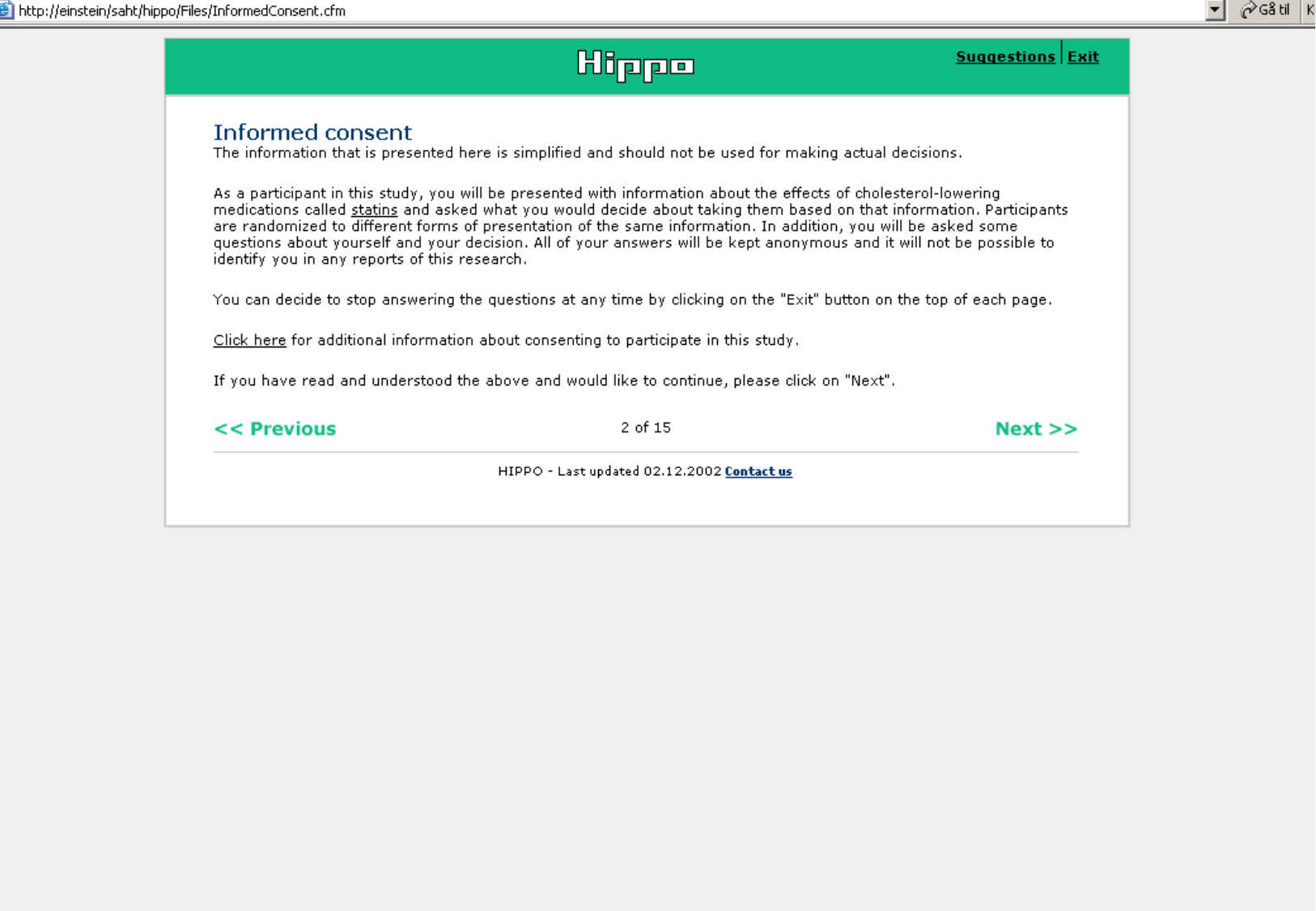

## Slide 3
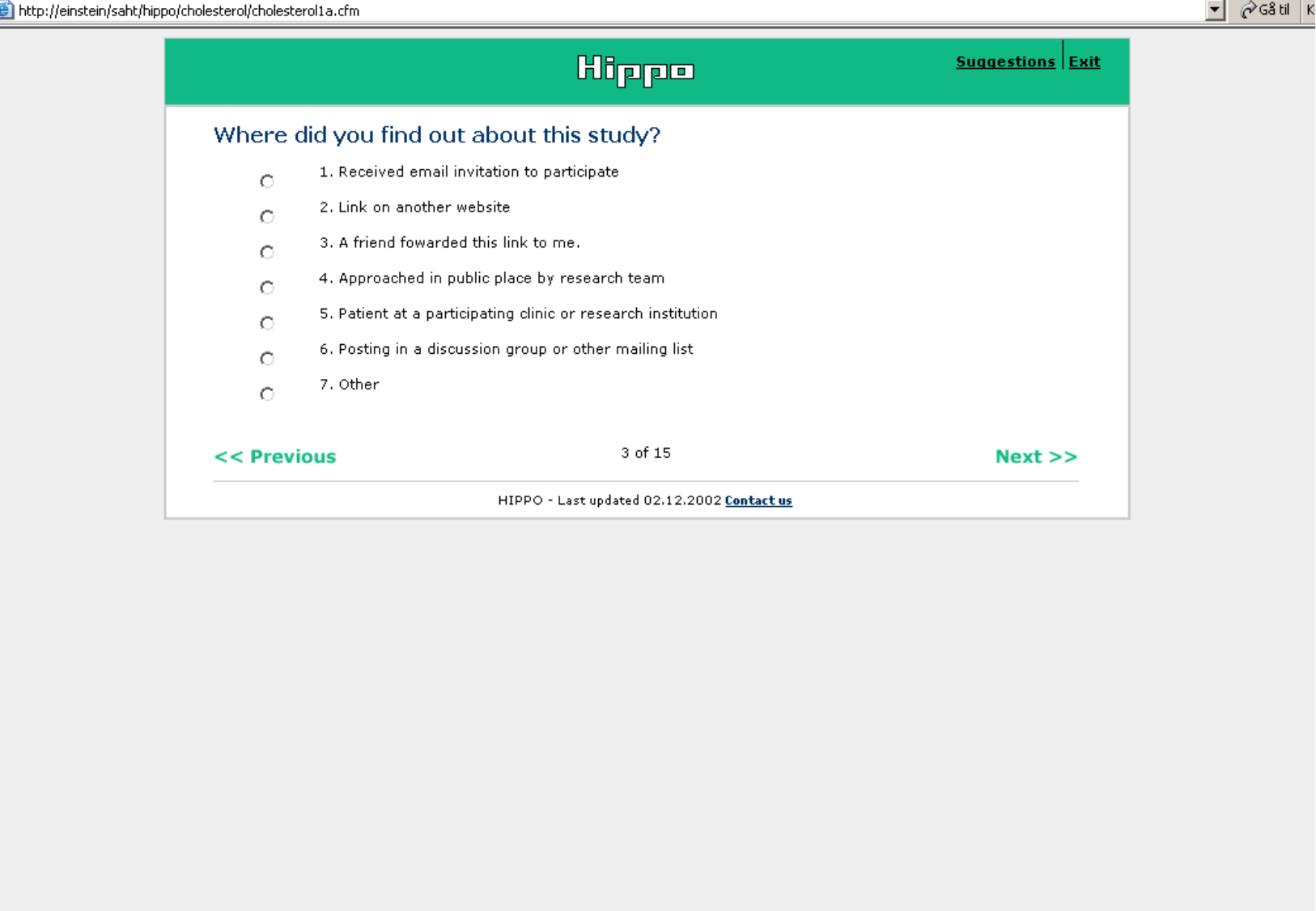

## Slide 4
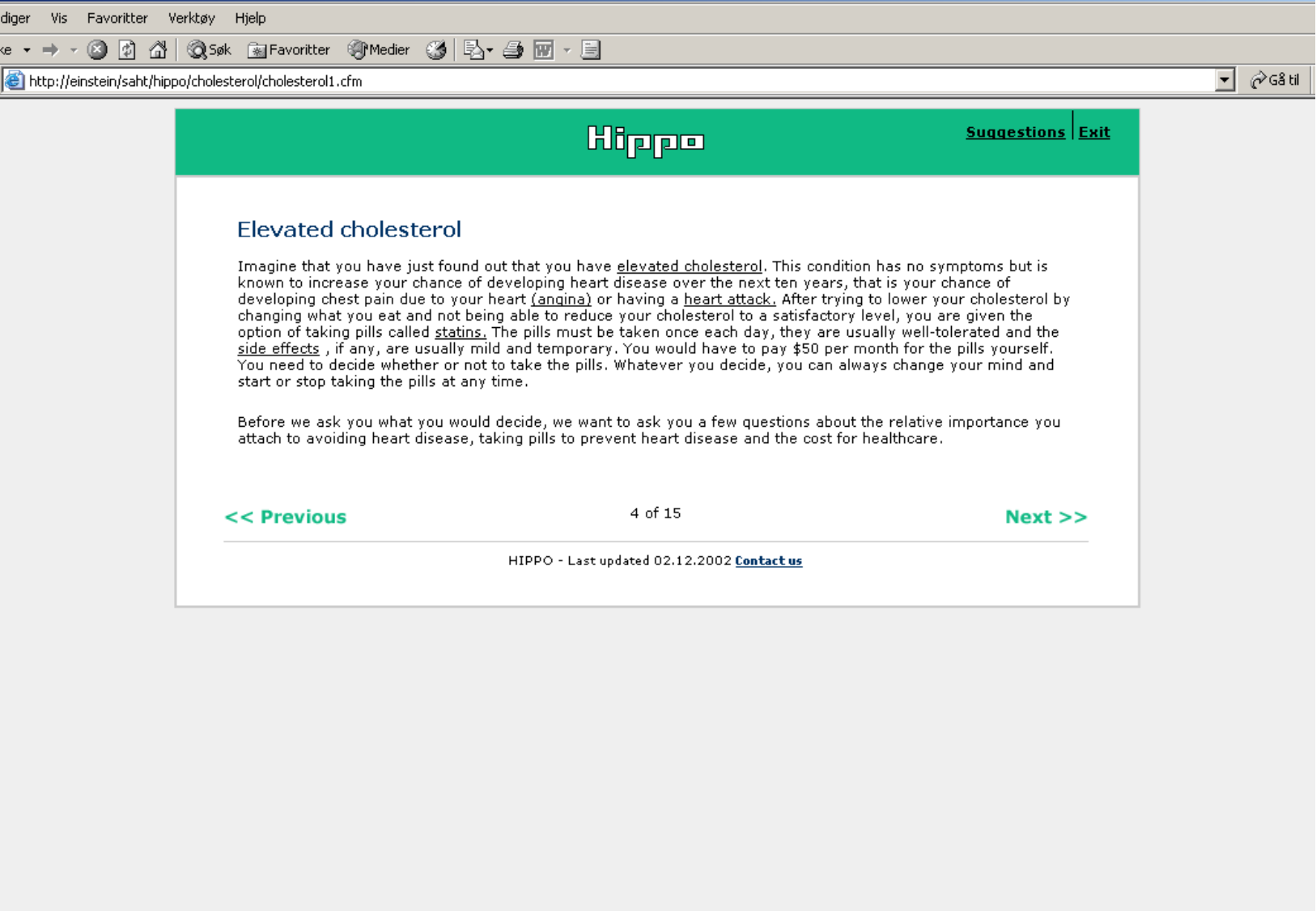

## Slide 5
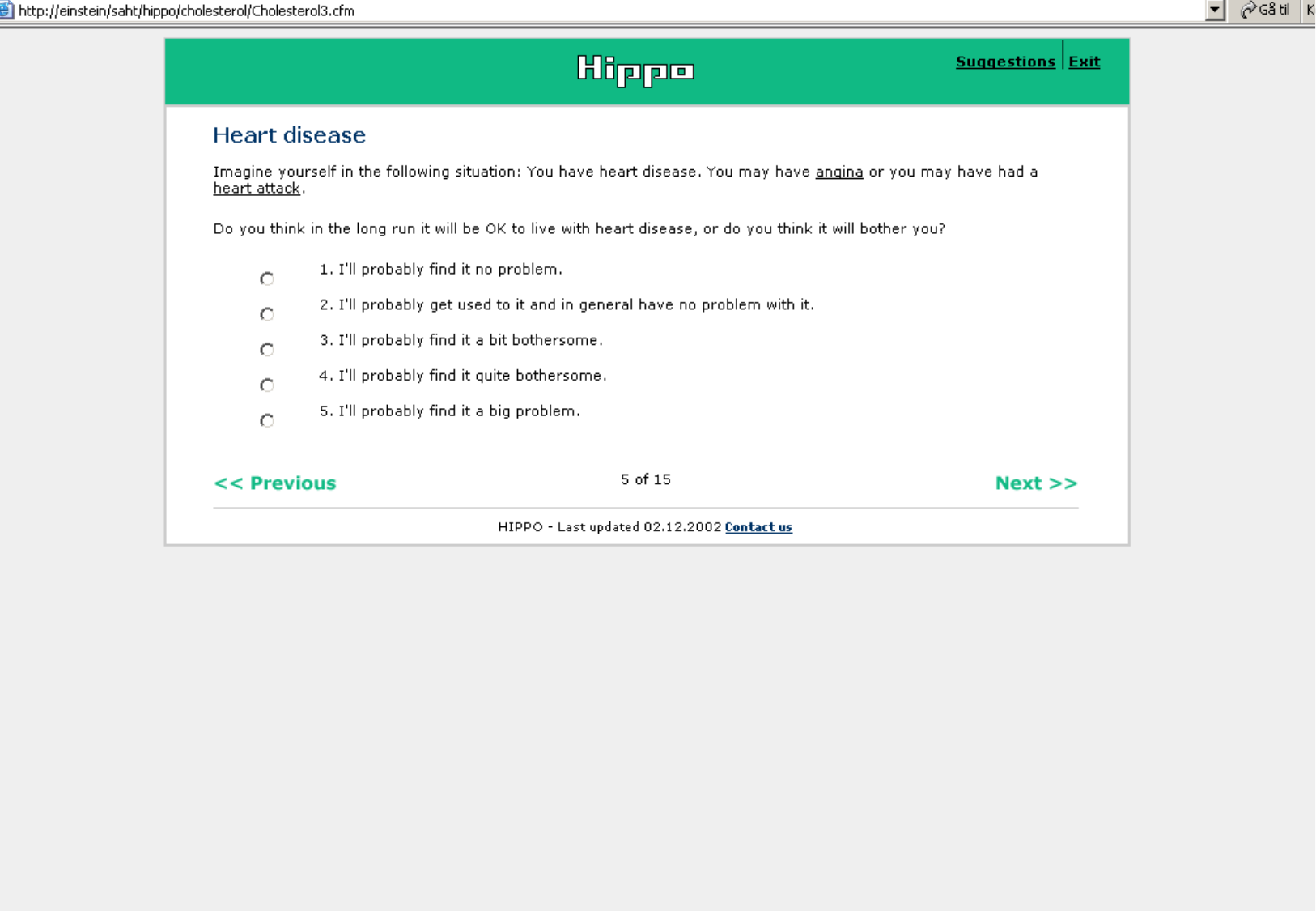

## Slide 6
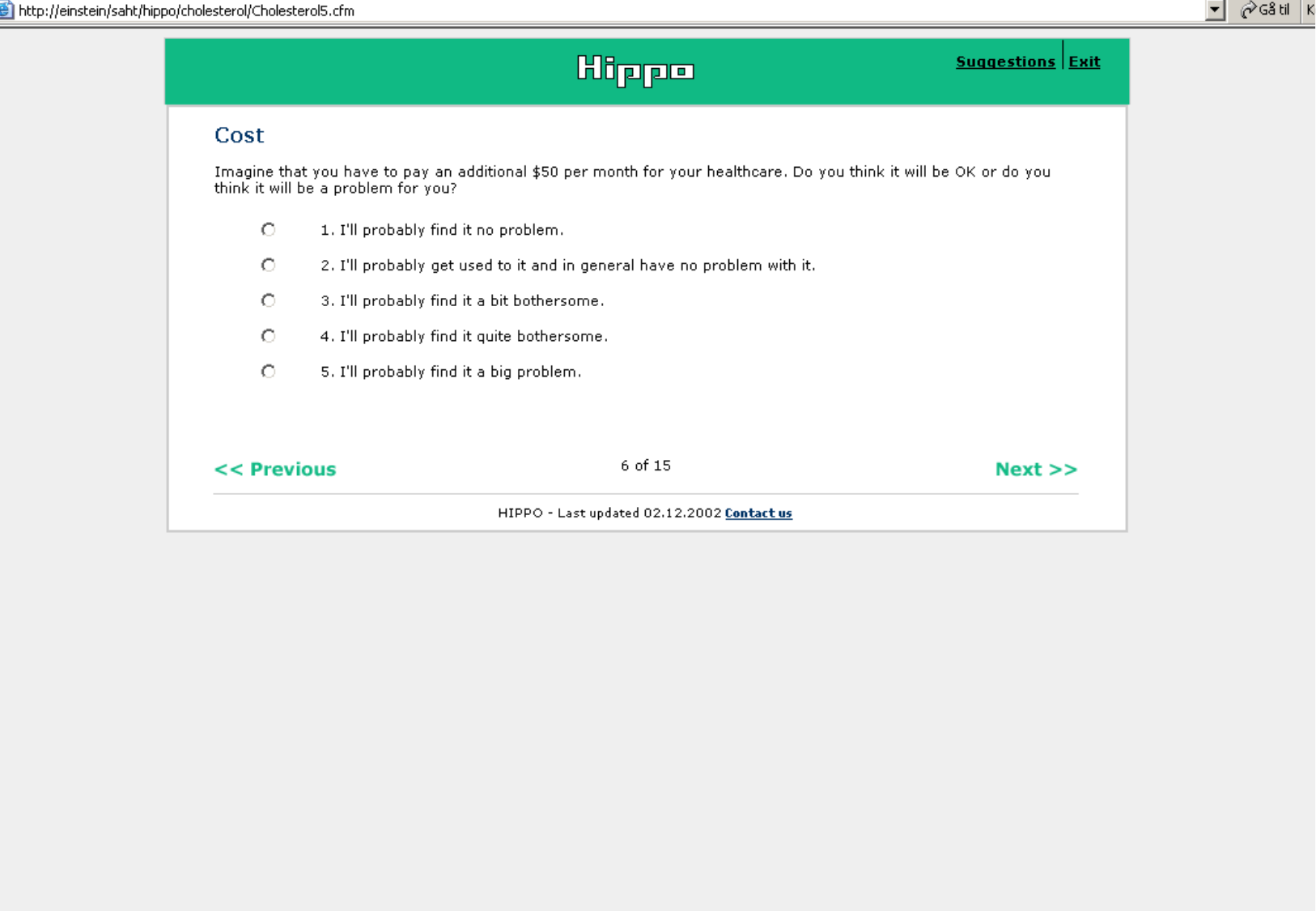

## Slide 7
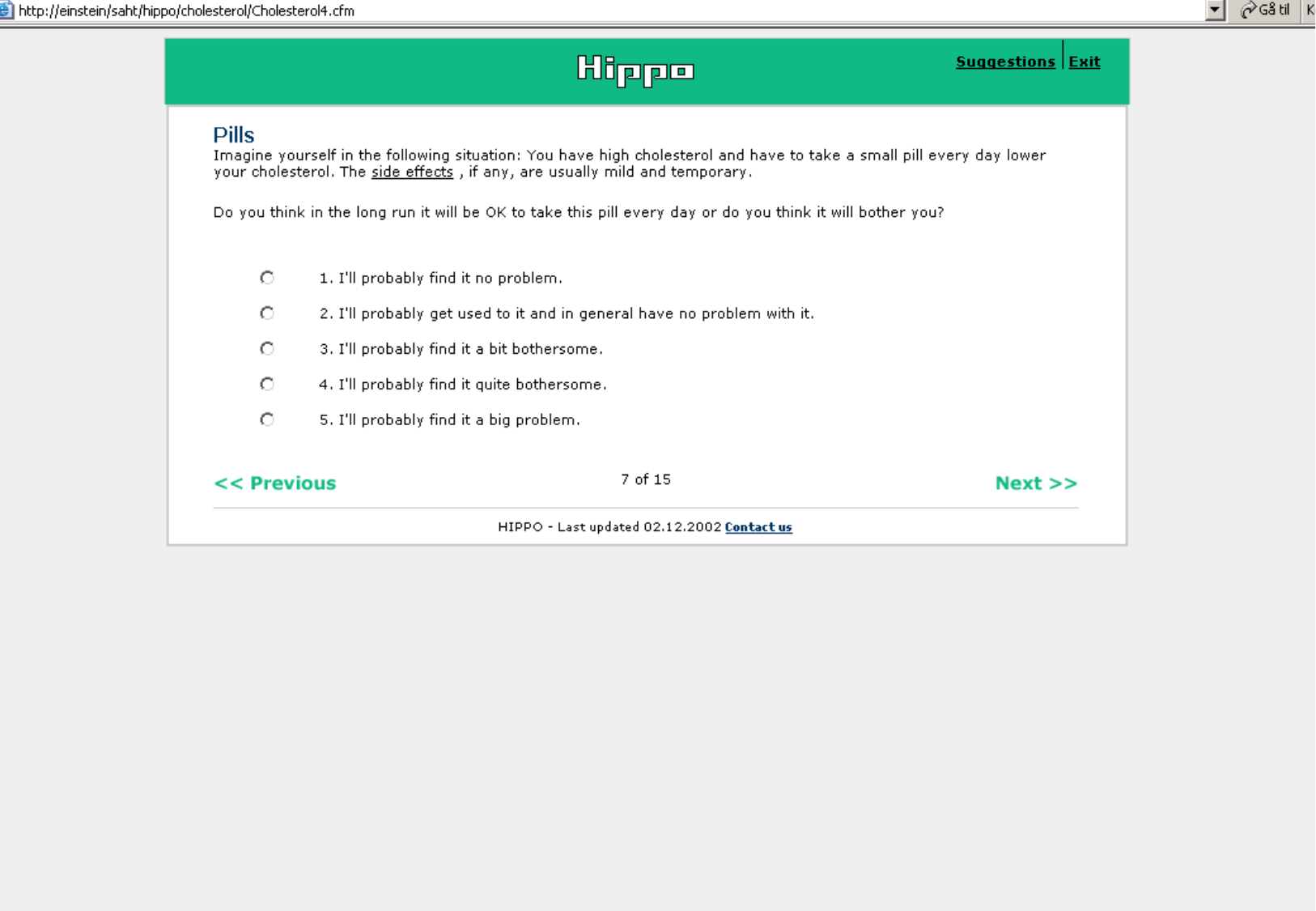

## Slide 8
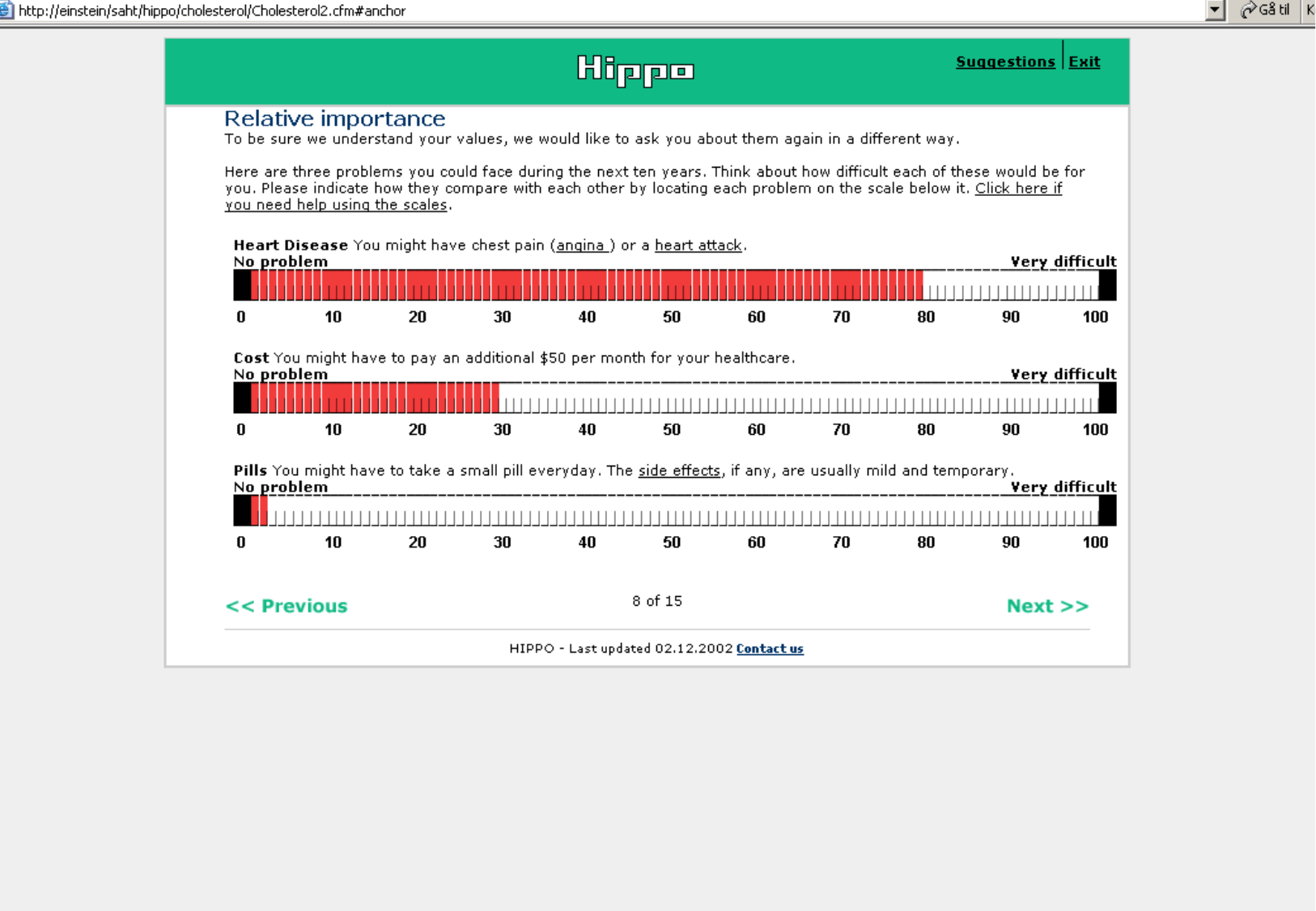

## Slide 9
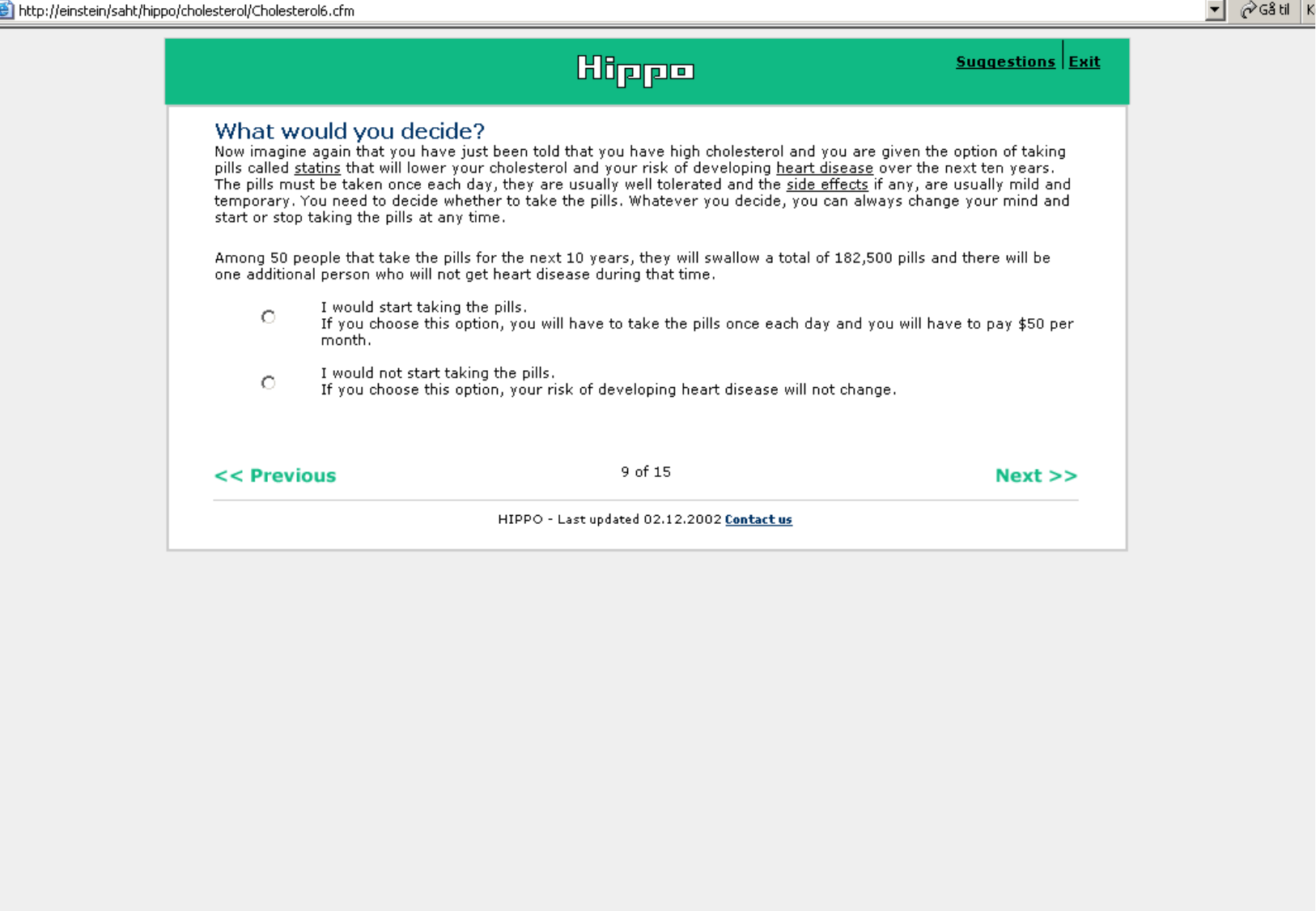

## Slide 10
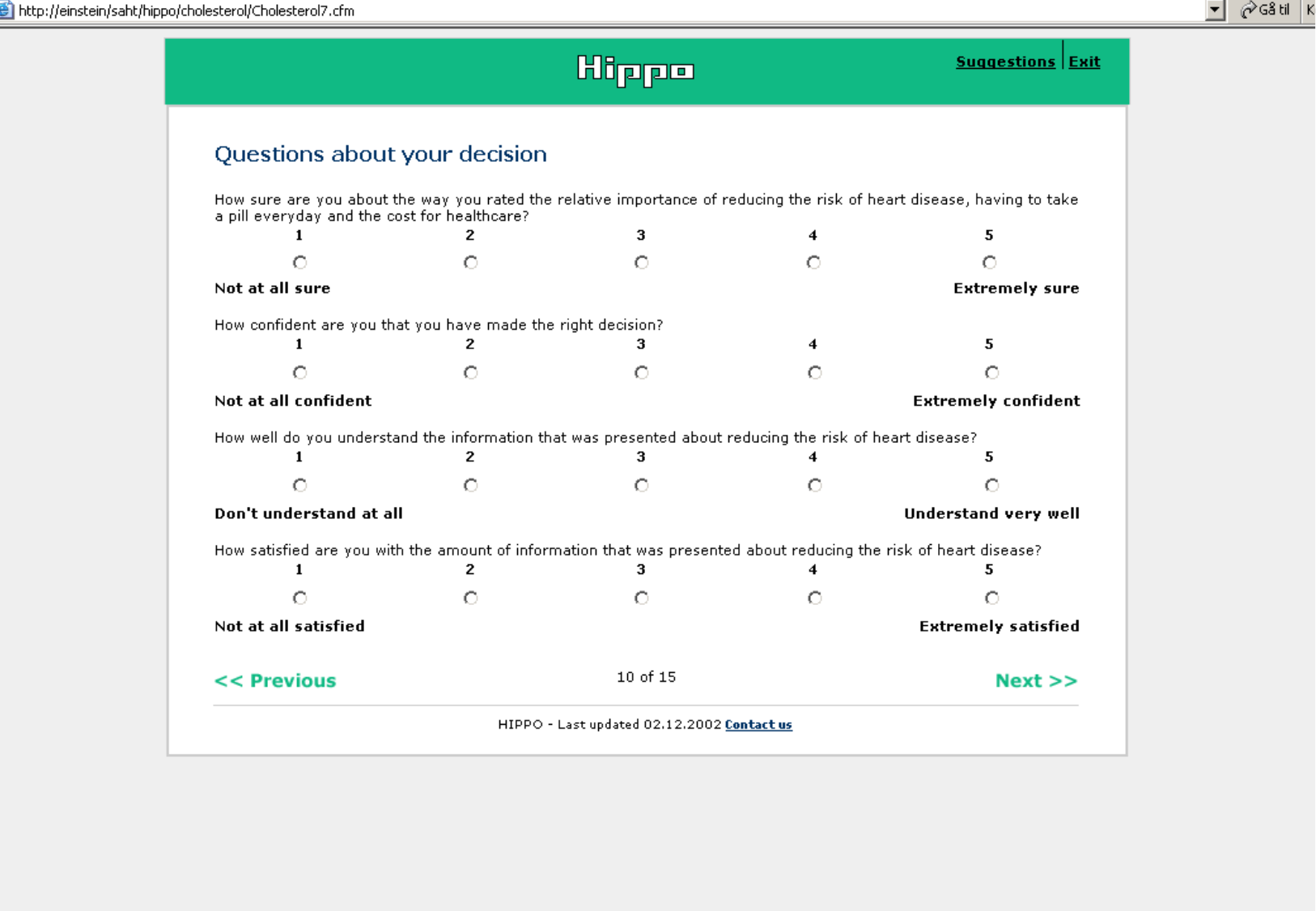

## Slide 11
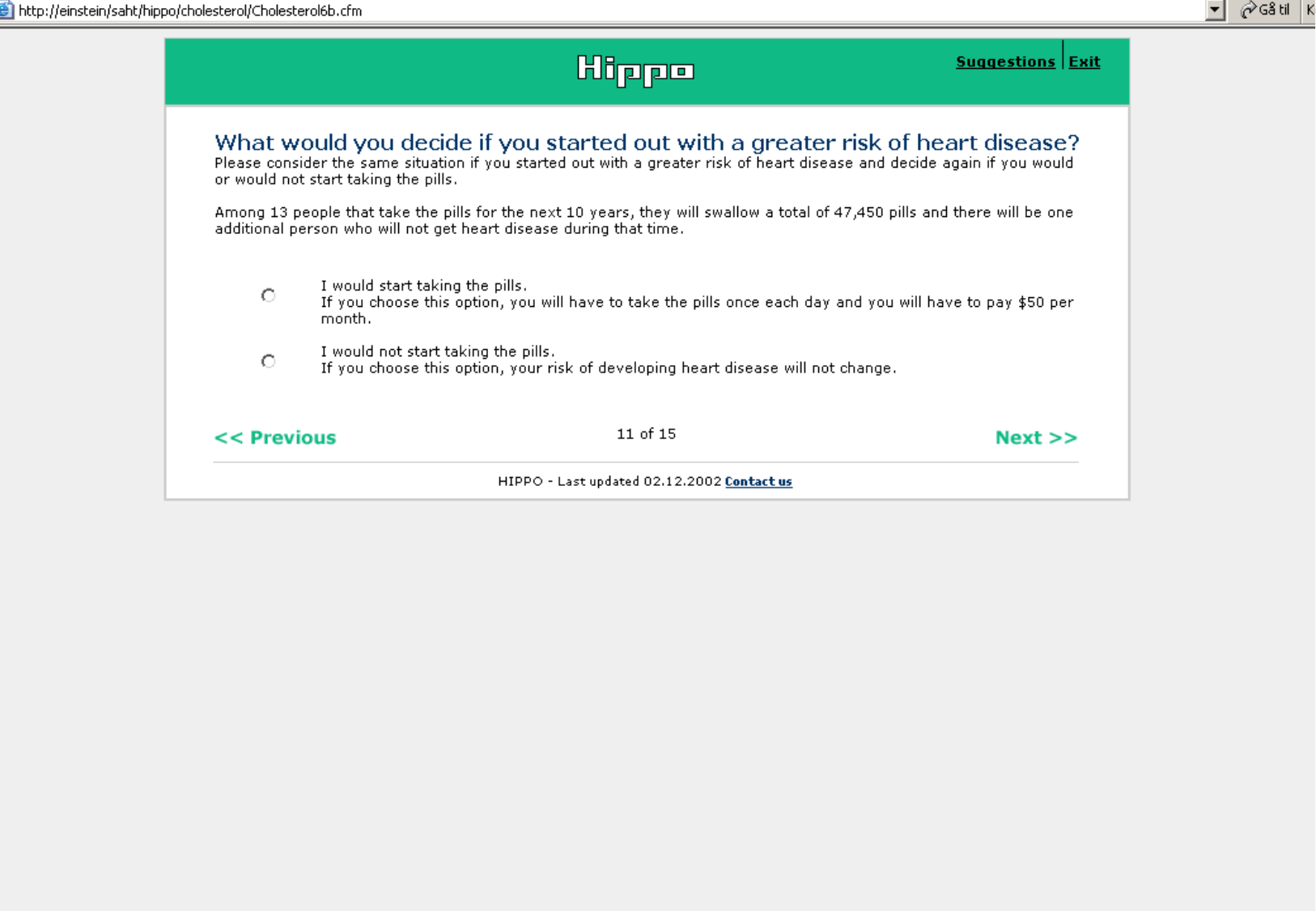

## Slide 12
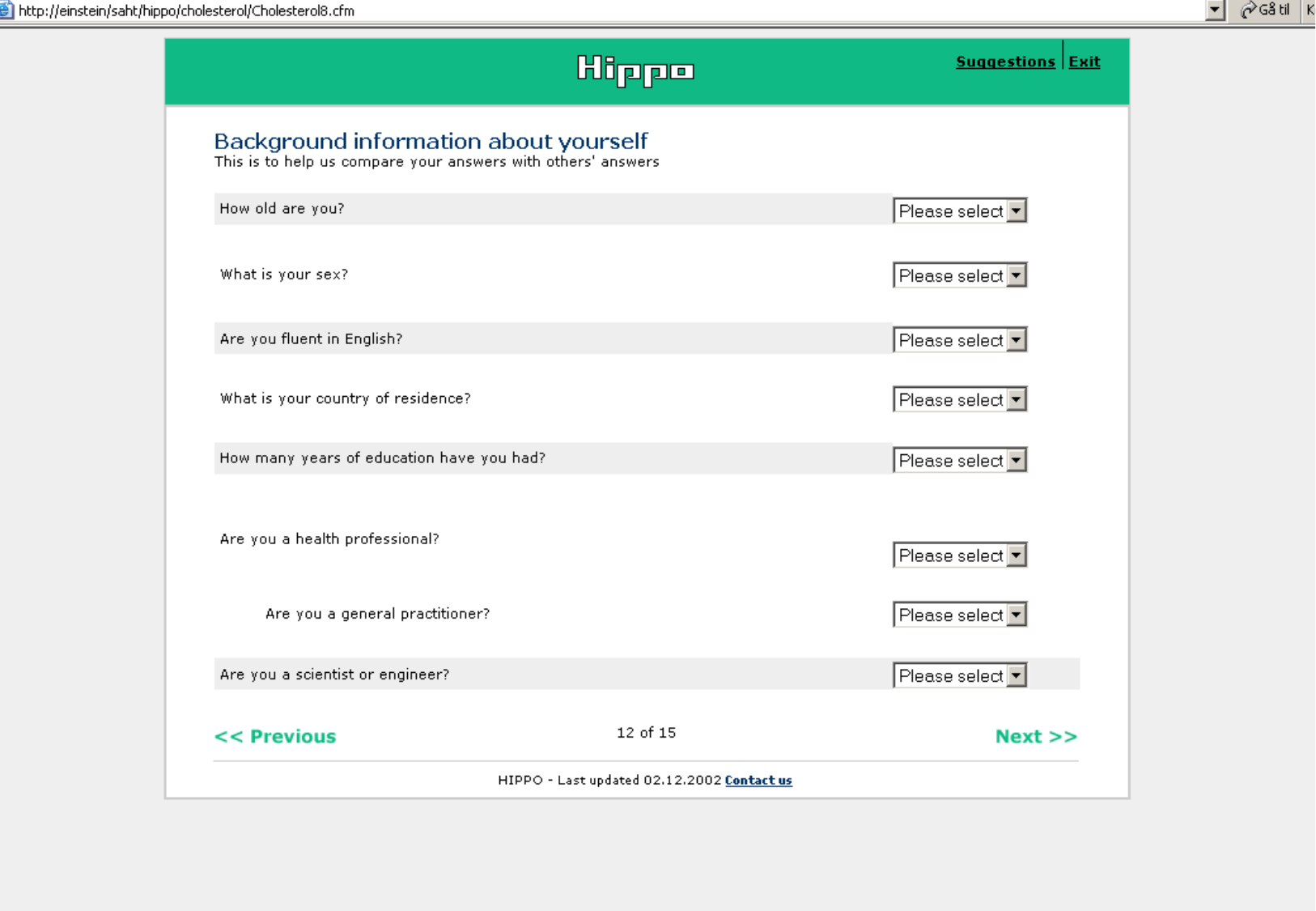

## Slide 13
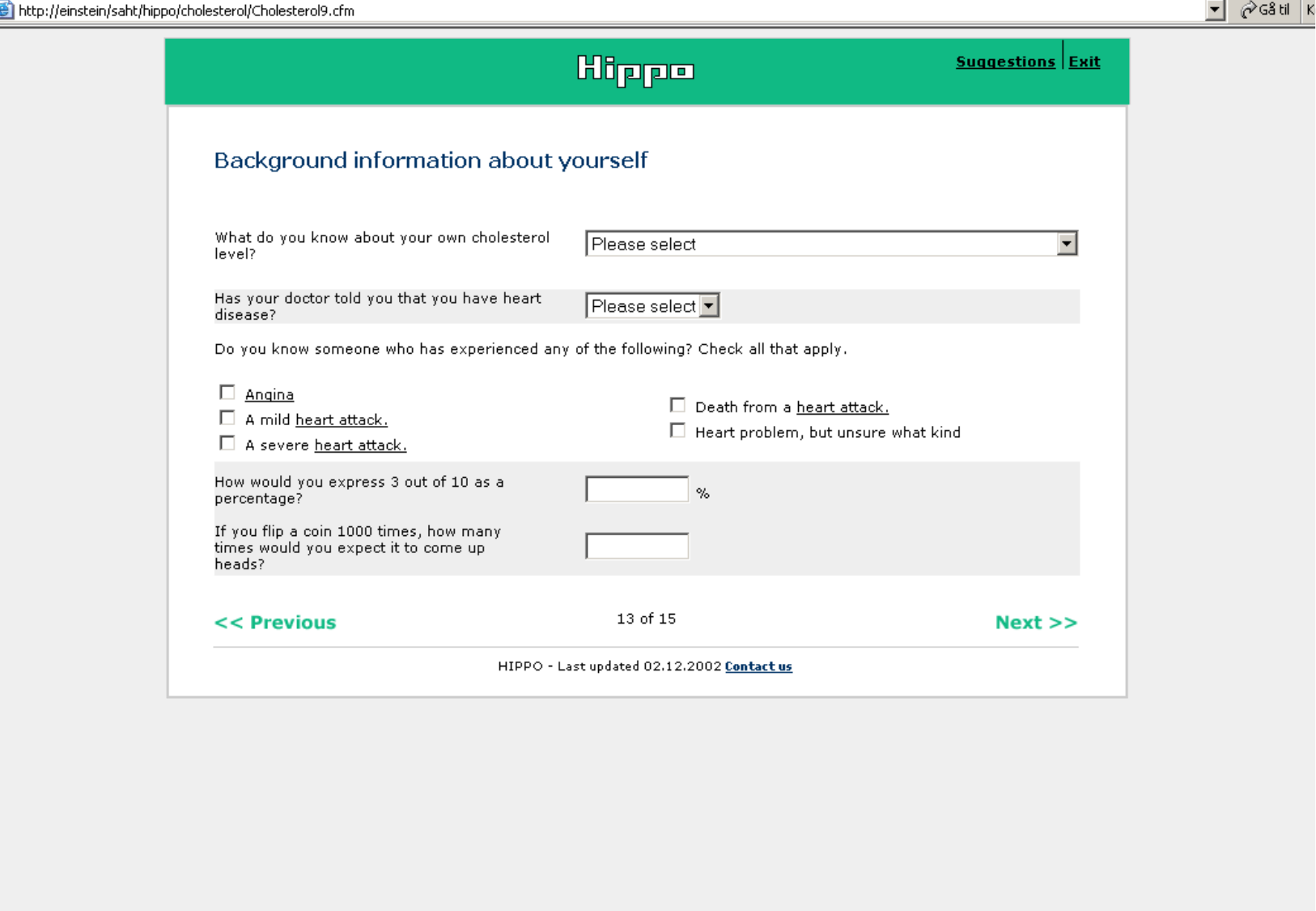

## Slide 14
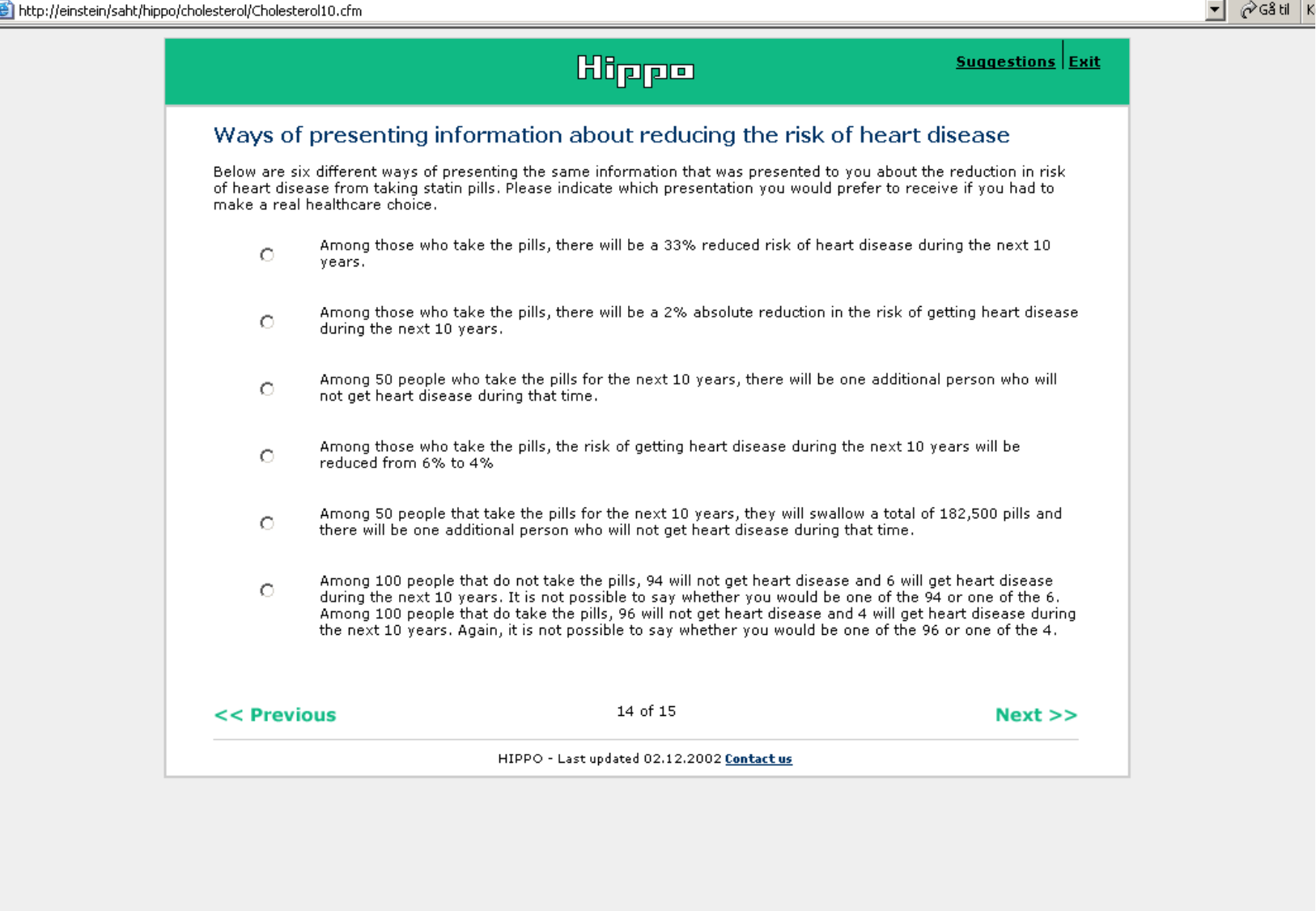

## Slide 15
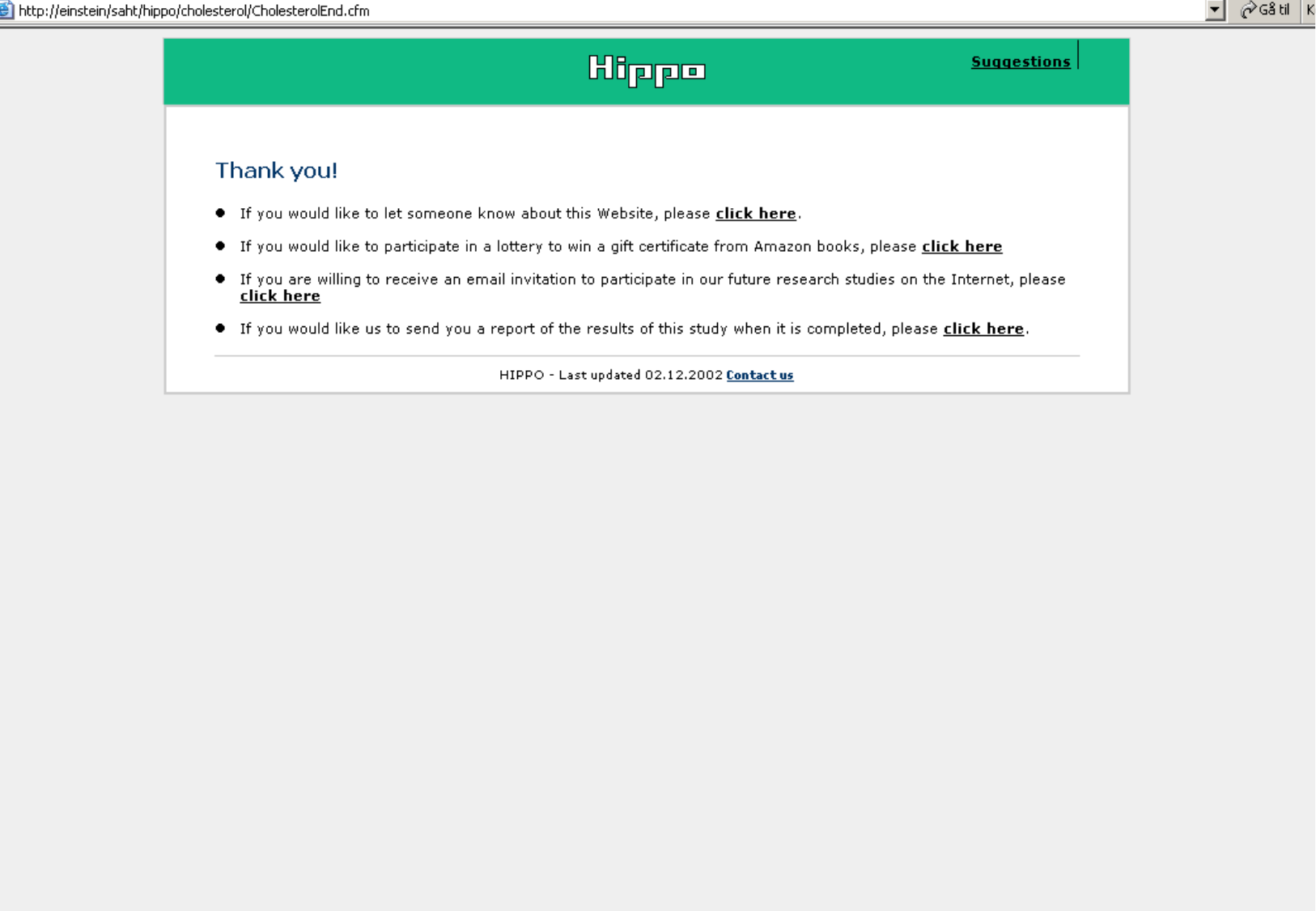

Supplement: Protocol S2 — Facsimile of HIPPO 1 webpages (0.88 MB PPT) [file pone.0003693.s006.ppt]
