# Supplementary material for: How Should the Impact of Different Presentations of Treatment Effects on Patient Choice Be Evaluated? A Pilot Randomized Trial
Source: PLoS One. 2008 Nov 24;3(11):e3693. doi: 10.1371/journal.pone.0003693 (PMC2585274; doi:10.1371/journal.pone.0003693)
Supplement: Appendix S1 — Methods (0.04 MB DOC) [file pone.0003693.s002.doc]

**Appendix**

Expected utility theory proposes that people making rational decisions should choose the option with the highest expected utility (EU), or, conversely, the lowest expected disutility, and prescribes the calculation of the EU for each decision option as the sum of the products of the utility (u) placed on each possible outcome of the option and the probability (p) of that outcome occurring:

EU = Σ p_i_ * u_i_

Because the study participants had been asked to indicate the relative magnitude of the *problem* of getting CHD (VAS_CHD_), of paying increased out-of-pocket costs for health care (VAS_Cost_), and of taking a pill everyday (VAS_Pills_), we calculated each participant’s expected “*disutility”* (PED) for each decision option, i.e. to start and to not start taking statin therapy, by substituting each participant’s elicited disutilities (D), i.e. VAS scores, for u in the above equation. The probability of getting CHD when taking statins (P_CHD(pills)_) is 4%, and when not taking statins (P_CHD(No pills)_) is 6%.

Each individual’s PED for the decision to take statins and the decision to not take statins is then:

PED(Taking pills) = P_CHD(pills)_ ּ(VAS_CHD_ + VAS_Pillls_ + VAS_Cost_) + (1-P _CHD(pills)_) ּ (VAS_Pillls_ + VAS_Cost_)

= P_CHD(pills) ‘_VAS_CHD_ + VAS_Pills_ + VAS_Cost_

PED(Not taking pills) = P_CHD(No pills)_ * VAS_CHD_

Using these PEDs, we calculated a relative importance score (RIS):

RIS EU_VAS = PED_No pills_ – PED_Pills_

Due to weighting by probabilities, the importance of having CHD had low impact on
RIS EU_VAS. We expected CHD to have more importance than taking a pill everyday or paying extra money for staying healthy, so we considered two alternative approaches for weighting the elicited values in order to estimate a RIS that would reflect this. To calculate RISPCA_VAS, we weighted each participant’s VAS scores with the first vector obtained from a principal component analysis (PCA) [1] that explained 52% of the variance. The interpretation of RISPCA_VAS is that it summarises the overall variability of the VAS values.

RISPCA_VAS = (PCA weight_CHD_ ∙ VAS_CHD_) + ((PCA weight_Pills_ ∙ VAS_Pills_) + (PCA weight _Cost_ ∙ VAS_Cost_) )

Logistic regression (LR) [2] was used to estimate coefficients (on the logit scale) for weighting each of the three VAS scores for calculating RISLR_VAS:

RISLR_VAS = (LR weight_CHD_ ∙ VAS_CHD_) + (LR weight_Pills_ ∙ VAS_Pills_) + (LR weight _Cost_ ∙ VAS_Cost_)

The values of RIS EU_VAS and RISLR_VAS increase as the magnitude of the disutility of getting CHD increases in relation to the summed disutilities for taking pills and paying an increased out-of-pocket cost for health care of US $50 (because the weights derived from both the PCA and LR for VAS_Pills_ and VAS_Cost_ were negative).

Finally, we calculated RIS ONE_VAS by weighting participants’ raw VAS-values equally, subtracting the downsides of statins from the benefit:

RIS ONE_VAS = (VAS_CHD_) - (VAS_Pills_) - (VAS_Cost_)

Four relative importance scores (RIS EU_CRS, RIS PCA_CRS, RIS LR_CRS, RIS ONE_CRS) were also calculated using CRS scores in place of VAS scores, thus producing eight distinct relative importance scores for each participant. These eight scores were then used in logistic regressions to compare the concordance between values and decision (to start statin therapy or not) across the six summary statistics. That is, for each of the eight RIS, the following logistic regression was fit:

logit(D) = β_0_ + β_1_(RIS)

where D represents the decision to start statin therapy. The resulting estimates for each of the four models using VAS and for the four models using CRS were compared and the c-statistic (measure of concordance), which is equal to the area under the receiver-operating characteristic (ROC) curve when the outcome is binary, was used to compare the discriminatory ability of the logistic regressions fitted for all RIS models for each summary statistic, i.e. 48 c-estimates [2]. A c-statistic of 1.0 indicates perfect accuracy, while a c-statistic of 0.5 indicates a non-discriminatory test. In addition, Hosmer-Lemeshow goodness-of-fit was calculated for each of the eight scores [2].

**Numeracy and salience**

The questions shown in Appendix S2 were used to assess numeracy (scored as 0, 1 or 2) and salience (scored from 0 to 4).

**References**

1. Mardia KV, Kent JT, Bibby JM (1982) Multivariate analysis. 3^rd^ printing. London: Akademic Press.
2. Hosmer DW, Lemeshow S (1992) Applied Logistic Regression, Second Edition. London: John Wiley & Sons Inc.
