# Supplementary material for: How Should the Impact of Different Presentations of Treatment Effects on Patient Choice Be Evaluated? A Pilot Randomized Trial
Source: PLoS One. 2008 Nov 24;3(11):e3693. doi: 10.1371/journal.pone.0003693 (PMC2585274; doi:10.1371/journal.pone.0003693)
Supplement: Protocol S1 — HIPPO 1. What is the effect of the summary statistic used to present the benefits of statins on decisions about whether to use them? (0.13 MB DOC) [file pone.0003693.s005.doc]

**What is the effect of the summary statistic used to present the benefits of statins on decisions about whether to use them?**

[Protocol]

Cheryl Carling, RN, MSc, Research Fellow^1^

Holger Schünemann, MD, PhD, Assistant Professor^2,4^

Jeph Herrin, PhD, Research Consultant^3^

Jan Arve Dyrnes, Consultant^1^

Shaun Treweek, PhD, Researcher^1^

Doris Tove Kristoffersen, MSc, Researcher^1^

Elie Akl, MD, Resident^2^

Gordon Guyatt, MD, Professor^4^

Phillip J. Deveraux, MD, Research Fellow^4^

Victor Montori, MD, MSc^4^

Andrew David Oxman, MD, Director^1^

1. Department of Health Services Research

Norwegian Directorate for Health and Social Welfare

PO Box 8054 Dep

N-0031 Oslo

Norway

1. Departments of Medicine and Social and Preventive Medicine

University of Buffalo

270 Farber Hall

3435 Main St

Buffalo, NY 14214

USA

1. Flying Butress Associates

PO Box 2254

Charlottesville, VA 22902

USA

1. Department of Clinical Epidemiology and Biostatistics

McMaster University

Room 2C12

Hamilton, Ontario, L8N 3Z5

Canada

Address for correspondence:

Cheryl Carling

Department of Health Services Research

Norwegian Directorate for Health and Social Welfare

PO Box 8054 Dep

N-0031 Oslo, Norway

Telephone +47 24 16 32 48

Fax +47 24 16 30 11

Email [cheryl.carling@shdir.no](mailto:cheryl.carling@shdir.no)

CONTENTS

[ABSTRACT 3](#__RefHeading___Toc8802286)

[BACKGROUND 5](#__RefHeading___Toc8802287)

[OBJECTIVES 7](#__RefHeading___Toc8802288)

[METHODS 8](#__RefHeading___Toc8802289)

[Study design 8](#__RefHeading___Toc8802290)

[Inclusion criteria 9](#__RefHeading___Toc8802291)

[Recruitment 9](#__RefHeading___Toc8802292)

[Pilot study 10](#__RefHeading___Toc8802293)

[Allocation 10](#__RefHeading___Toc8802294)

[Data collection 11](#__RefHeading___Toc8802295)

[Analysis 11](#__RefHeading___Toc8802296)

[REFERENCES 17](#__RefHeading___Toc8802300)

[Sample size 13](#__RefHeading___Toc8802297)

[Descriptive statistics 14](#__RefHeading___Toc8802298)

[Secondary comparisons 15](#__RefHeading___Toc8802299)

[ATTACHMENT 2 – Alternative analyses 18](#__RefHeading___Toc8802301)

[ATTACHMENT 2 – Risk presentations 19](#__RefHeading___Toc8802302)

SUPPORTING INFORMATION S2.1 - Facsimile of HIPPO webpages…………………………………………………

## ABSTRACT

Background: For patients, healthcare professionals and policy makers to make informed choices about healthcare they must have information about the effects of interventions that is valid and understandable. The manner in which this information is presented affects how it is understood and subsequent decisions. The Health Information Project: Presentation Online (HIPPO) is designed to improve communication of information about the effects of healthcare based on randomized trials of alternative ways of presenting this evidence. Expected utility theory suggests that people making rational decisions should choose the option with the highest expected utility. The utility of outcomes, such as different health states, is usually expressed as being between zero and one (e.g. with death having a value of zero and a full healthy life having a value of one). Expected utility theory has been questioned for a number of reasons, including problems with how utilities are measured and observations that people often do not, in fact, choose to maximize their utilities.

Objective: The primary objective of this study is to determine which ways of presenting information about the reduction in risk of cardiovascular disease (CVD) result in decisions that are most consistent with what individuals considering starting statins for the primary prevention of CVD would be expected to decide based on their expected utilities.

Methods: The study is an Internet-based randomized trial in which participants will be randomized to one of six ways of presenting information about CVD risk reduction. Participants will be recruited through invitations to participate by email, links to the study website from Web pages that provide health information, Internet surfers who come across the HIPPO Website, being approached in public areas, Internet discussion groups of general practitioners.

Conclusions: The results of this study will help to inform decisions about how best to communicate the benefits of statins to healthcare professionals (general practitioners), individual patients and the general public.

## BACKGROUND

For patients, healthcare professionals and policy makers to make informed choices about healthcare they must have information about the effects of interventions that is valid and understandable. The manner in which this information is presented affects how it is understood and subsequent decisions.^1,2^  The objective of the Health Information Project: Presentation Online (HIPPO) is to improve communication of information about the effects of healthcare based on randomized trials of alternative ways of presenting this evidence.

This is the first HIPPO trial. Information about the effects of drugs used to decrease cholesterol blood levels was chosen for this study because it is a common problem and high quality evidence is available on the effects of cholesterol-lowering drugs (statins).^3^ The results of this study will help to inform decisions about how best to communicate the benefits of statins to healthcare professionals (general practitioners), individual patients and the general public.

This study is also a pilot study intended to test the methods that we will use in future HIPPO studies. We have therefore selected comparisons for which there exists prior research evidence. Two systematic reviews have concluded that the use of the relative risk reduction (RRR) to express the relationship between two probabilities results in individuals having a larger estimate of that relationship and being more likely to decide to treat compared with the use of an absolute risk reduction (ARR) or number needed to treat (NNT).^1,2^ None of the studies included in these reviews has investigated the relationship between the summary statistic that is used and the extent to which decisions are congruent with “expected utilities”.

Expected utility (or “expected value”) can be defined as follows: Under uncertainty where there is a p% chance of an outcome (X), say having angina or a heart attack, the expected utility is equal to pּU_x_ + (1-p)ּU_not x_ where U_x_ represents the desirability (utility value) of the outcome and U_not x_ is the desirability of the alternative outcome (good health). If there is a p% chance of X and a q% chance of another outcome (Y), then the expected value is equal to [pּU_x_ + (1-p)ּU_not x_ + qּU_y_ + (1-q)ּU_not y_]. Expected utility theory suggests that people making rational decisions should choose the option with the highest expected utility.^4^ The utility of outcomes, such as different health states, is usually expressed as being between zero and one (e.g. with death having a value of zero and a fully healthy life having a value of one, although some people consider some health states to be worse than death).

Expected utility theory has been questioned for a number of reasons, including problems with how utilities are measured and observations that people often do not, in fact, choose to maximize their utilities.^5-9^ Nonetheless, it can still be argued that as the expected utility for a decision, such as to take statins, increases one would expect that, on average, increasing proportions of people would choose to take the pills, if they are well informed. This argument does not depend on every individual choosing to maximize her utilities. Some people may make decisions based on other factors and it is difficult to accurately measure people’s utilities. Nonetheless, one would expect some degree of correlation between the expected utility of deciding to do something, such as taking pills, and the proportion of people who decide take the pills. The assumption underlying this study is that ways of presenting information about the effects of healthcare that result in decisions that are most consistent with individuals’ subjective expected utilities are best.

## OBJECTIVES

The primary objective of this study is to determine which of the following ways of presenting information about the reduction in risk of cardiovascular disease (CVD) results in decisions that are most consistent with what individuals considering starting statins for the primary prevention of CVD would be expected to decide based on their expected utilities:

1. RRR - Among those who take the pills there will be a 33% reduced risk of heart disease during the next 10 years.
2. ARR - Among those who take the pills, there will be a 2% absolute reduction in the risk of getting heart disease during the next ten years.
3. NNT – Among 50 people that take the pills for the next ten years there will be one additional person who will not get heart disease during that time.
4. Event rates - Among those who take the pills the risk of getting heart disease during the next ten years will be reduced from 6% to 4%.
5. TNT (tablets needed to be taken)^10^ – Among 50 people that take the pills for the next ten years, they will swallow a total of 182,500 pills and there will be one additional person who will not get heart disease during that time.
6. Whole numbers^11^ - Among 100 people who do not take the pills, 94 will not get heart disease and six will get heart disease during the next 10 years. It is not possible to say whether you would be one of the 94 or one of the six. Among 100 people who take the pills, the number who will not get heart disease in the next ten years will increase from 94 to 96 and the number who will get heart disease during the next 10 years, will decrease from 6 to 4.. Again, it is not possible to say whether you would be one of the 96 or one of the four.

Additional questions that this study will address are:

1. What is the effect of these different ways of presenting information about CVD risk reduction on understanding, confidence and satisfaction?
2. Which of the following two ways of eliciting the relative importance of the consequences of starting statins for the primary prevention of CVD gives “expected utilities” that are most congruent with what individuals decide?

- Visual analog scales (VAS’s)
- Likert-type rating scales (LTRS)

1. Are answers to the primary question and the two questions above consistent across patients (the general public) and general practitioners (GP’s)?
2. Are answers to the first three questions consistent across different countries (Norway, USA, Canada and Germany)?

## METHODS

### Study design

The study is a Web-based randomized trial in which participants will be randomized to one of six ways of presenting information about CVD risk reduction listed above. The relative importance of the consequences of starting statins will be elicited in two ways (VAS’s and LTRS’s) for all of the participants. The order in which this is done will be randomized.

A printout of the Webpages that will be used is attached [Available as a supporting PowerPoint file, Figure S3.1 Facimile HIPPO Webpages for protocol]. Upon logging into the study Website, participants will be given information about the study and asked to give informed consent to participate. They will be presented with a brief scenario in which they are asked to imagine that they have elevated cholesterol and are given the option of taking pills that lower cholesterol (statins). The relative importance of CVD risk reduction, taking pills and costs will be elicited using VAS’s and LTRS’s. They will then be presented with information about the effect of statins on the risk of heart disease, using one of the presentations listed above, the need to take a pill each day and the out-of-pocket costs; and asked whether they would decide to take the pills or not. Baseline risk will then be altered in the direction that shifts the expected utilities towards the opposite decision; i.e. the baseline risk will be increased for respondents who decide not to take the pills and decreased for those who decide to take the pills. Participants will then be asked what they would decide again. After this they will be asked questions about their decision and about themselves and, finally, they will be asked which of the six ways of presenting CVD risk reduction they would prefer.

### Inclusion criteria

To be included in the trial participants must be > 18 years old and literate in one of the following languages English, French, German, Norwegian.

### Recruitment

Participants will be recruited in the following ways:

- Invitations to participate will be sent to individuals by email in the US. The email addresses will be obtained through a vendor who has enlisted opt-in respondents for issues regarding health.
- Patients at a participating research facility will be given the opportunity to participate.
- Three additional means of recruiting participants will be used to assess the potential to recruit participants without sending out invitations by email and to compare the extent to which there are differences in results between participants recruited in these different ways:
  - Links to the study Website will be added to various Web pages that provide health information.
  - Internet surfers who come across the HIPPO Website will be invited to participate.
  - People will be approached in public areas, such as a shopping mall, and invited to participate using a computer that will be provided and with assistance, if it is needed.
- GPs will be contacted through Internet discussion groups or by email and invited to participate.

Potential participants will be offered options to participate in a lottery for a $100 gift certificate and to receive a report of the results of the study.

### Pilot study

Prior to conducting the study, we will conduct a pilot study in which we will only recruit people in the USA. The aims of the pilot study will be to: ensure that recruitment and data collection function as anticipated; obtain data to more accurately estimate the necessary sample size; and determine how best to analyze the data.

### Allocation

Allocation to one of the six presentations will be determined by block-randomization. We have created a sequence of 600 presentation assignments by generating 100 blocks of the 6 presentations using [http://www.randomization.com](http://www.randomization.com/) (Randomization plan created on 17 April 2002, 16:03:41. To reproduce this plan use the seed 11049). This sequence will be looped and repeated until an adequate sample size is achieved.

The order in which we elicit the relative importance of the consequences of starting statins, VAS’s versus LTRS’s) will also be allocated by block-randomization.

### Data collection

Participants will enter responses directly by responding to the questions on the HIPPO website. The data generated will be stored anonymously in a database. Only completed records will be used in the main analyses.

### Analysis

We have the following variables:

U_CVD_ = utility of having heart disease; i.e. 1 – how difficult it would be to have cardiovascular (“heart”) disease on a scale from 0 to 1; 1 equals the most difficult

U_Pill_ = utility of having to take a pill each day; i.e 1 – how difficult it would be to have to take a pill each day on a scale from 0 to 1; 1 equals the most difficult

U_Cost_ = utility of not having to pay for pills; i.e. 1 – how difficult it would be to have to pay an additional amount of money each month for equivalent to the out-of-pocket cost of taking a statin on a scale from 0 to 1; 1 equals the most difficult

P_P_ = probability of having heart disease with taking pills

P_N_ = probability of having heart disease without taking pills

Using these variables we can calculate the expected utility (EU) for a decision to take pills:

EU(taking pills) = PPּU_CVD_ + (1 - P_P_)ּ(1-U_CVD_) – (1-U_Pill_) – (1-U_Cost_)

Operationally this transforms to:

EU(taking pills) = PPּ(1-VAS_hrt) + (1 - P_P_)ּ(VAS_hrt) – (VAS_pill) – (VAS_cost)

or not to take pills:

EU(not taking pills) = PNּU_CVD_ + (1 - P_N_) ּ (1-U_CVD_)

Operationally this transforms to:

EU(not taking pills )= PNּ(1-VAS_hrt) + (1 - P_N_)ּ(VAS_hrt)

and the difference between these (A):
A = EU(taking pills) – EU(not taking pills)

The difference in expected utilities can range from positive values (in favour of taking pills) to negative values (in favour of not taking pills).

If G is used to represent the presentation group to which a participant is randomised and D represents the decision to take pills or not, then the relationship between D and A can be modelled as follows:

(Eq 1)          logit(D) = β_0_ + β_1_A

To examine the relationship between the model of what participants might be expected to decide based on the expected utilities of the two options (Eq 1) with how the information was presented, Eq 1 will be fit for each group G. We will then compare the fit between different groups by comparing the R^2^ between the different groups.

Using a maximum-likelihood model will allow us to adjust for other factors (age, gender, profession) in a natural way. This allows us to test for differences in professional background (scientists or engineers vs GPs or other health professionals vs others), and differences across countries (Canada vs Germany vs Norway vs USA). To the extent that any of these factors are confounders, adjusting for these factors would improve the precision of our model.

### Sample size

Random variation in choices can be expected to be smaller the better risk information is understood. So we will expect a stronger association (larger R^2^) between the expected utility model (A) and what participants decide (D) the better the information about the risk of heart disease is understood. Turning this around, a significantly stronger association between the model and what participants decide for one presentation (G_1_) compared with another (G_2_) suggests that the first presentation was better understood than the second.

The detectability of a difference in magnitude between population R’s is not a simple function of the difference.^10^ That is, if we were to define j = R_1_ – R_2_ and try to use j as the effect size, the detectability of j under fixed conditions of α (level of significance) and sample size n, is not constant, but depends on where along the R scale the difference j occurs. The Fisher Z transformation of R provides a solution to this, and the effect size index q = Z_1_ – Z_2_ gives values whose detectability does not depend on whether the Z’s are both small or both large. Cohen considers q = 0.10 as a small effect size, and the following pairs of R’s illustrate this amount of difference: (0.00;0.10), (0.20; 0.29), (0.40; 0.48), (0.60; 0.66), (0.80,0.83), (0.90;0.92), (0.95;0.96). A medium effect size is q = 0.30 and the following pairs of R’s illustrate this amount of difference: (0.00;0.29), (0.20; 0.46), (0.40; 0.62), (0.60; 0.76), (0.80,0.89), (0.90;0.94), (0.95;0.97). A large effect size is when q = 0.50. This is illustrated by the following pairs of R’s: (0.00;0.46), (0.20; 0.61), (0.40; 0.73), (0.60; 0.83), (0.80,0.92), (0.90;0.96), (0.95;0.98).

As we have no prior information about the size of R within any of the information groups, we will conduct a pilot study. The number of subjects to be included in each group in the pilot study is based on power calculations for detecting a medium and a somewhat larger effect size (assuming equal size of the groups):

| q | α (2-sided test) | Power | N |
| --- | --- | --- | --- |
| 0.30 | 0.05 | 0.70 | 140 |
|  |  | 0.75 | 157 |
|  |  | 0.80 | 177 |
|  | 0.10 | 0.70 | 108 |
|  |  | 0.75 | 123 |
|  |  | 0.80 | 140 |
| 0.40 | 0.05 | 0.70 | 80 |
|  |  | 0.75 | 90 |
|  |  | 0.80 | 101 |
|  | 0.10 | 0.70 | 62 |
|  |  | 0.75 | 70 |
|  |  | 0.80 | 80 |

As can be seen from the table above, the required sample size varies from 80 to 140 per group. However, if we manage to recruit a minimum of 100 subjects into each group, we are likely to obtain estimates for the difference in R’s in addition to estimates of the R for each group.

The total sample size that is needed will be calculated based on the results of the pilot study. Because we are not certain how best to analyse the data, we will also explore alternative ways of analysing the results (see Attachment 2) and, if needed, adjust the sample size calculations to take account of this.

### Descriptive statistics

The results will be summarised in tables by means, standard deviations, counts and percentages as appropriate for the each variable for each (presentation) group of participants and for all participants. Density distributions will be used to present the VAS results, which are continuous. Bar charts will be used to present the LTRS results, which are categorical. Graphical displays will also be used to compare the VAS and LTRS results.

### Secondary comparisons

Because participants will only be recruited from the general public in the USA for the pilot, it will be possible to compare the use of VAS’s and LTRS’s, but not to conduct the other secondary comparisons with data from the pilot study. The analysis comparing the use of VAS’s to LTRS’s will be similar to the analysis comparing different presentations. We expect a stronger association (larger R^2^) between the expected utility model (A) and what participants decide (D) the better the way of eliciting the relative importance of the consequences of starting statins gives values that are congruent with the participants true values. Turning this around, a significantly stronger association between the model and what participants decide for one way of eliciting values (VAS’s) compared with the other (LTRS’s) would suggest that the first way was better than the second.

#### ETHICS

The interventions in this study, alternative ways of presenting information on risk reduction, and asking the participants to make a hypothetical decision, are non-invasive and harmless. The same information presented in this study is widely available and is presented in many different ways, including those used in this study.^10^

Participants are informed on the consent screen that they can leave the study at any time, and they are given the option of having any data that they might have entered deleted.

Confidentiality of data entered by participants is ensured by not collecting any information that would make it possible to identify the participants. If participants fill out a form to request a report of the study results or they choose to participate in the lottery for a $100 gift certificate, their email or postal addresses will not be stored in the same database as their responses to the study questions.

## REFERENCES

1. McGettigan P, Sly K, O’Connell D, Hill S, Henry D. The effects of information framing on the practices of physicians. J Gen Intern Med. 1999; 14:633-42.
2. Herrin J, Schünemann H, Oxman AD, Vist G, Olsen K. Presentation of empirical evidence about health (Cochrane Review). In: The Cochrane Library. Oxford: Update Software. Under revision.
3. Ebrahim S, Davey Smith G, McCabe C, Payne N, Pickin M, Sheldon T A, et al. What role for statins: a review and economic model. Health Technology Assessment 1999, 1-91.
4. Von Neumann J,Morgenstern O. Theory of Games and Economic Behavior. New York: Wiley, 1944.
5. Schoemaker PJH. The expected utility model: its variants, purposes, evidence and limitations. J Economic Literature 1982; 20:529-35.
6. Llewellyn-Thomas H, Sutherland HJ, Tibshirani R, et al. The measurement of patients’ values in medicine. Med Decision Making 1982; 2:449-62.
7. Hellinger FJ. Expected utility theory and risky choices with health outcomes. Med Care 1989; 27:273-9.
8. Frisch D, Clemen RT. Beyond expected utility: rethinking behavioral decision research. Psychol Bull 1994; 116:46-54.
9. Schwartz S, Griffin T. Medical Thinking: the Psychology of Medical Judgment and Decision Making. New York: Springer-Verlag 1986; 13.
10. Skolbekken JA. Communicating the risk reduction achieved by cholesterol reducing drugs. BMJ 1998; 316:1956-8.
11. Hollnagel H. On the language of risk in the medical consultation [Danish]. Practicus 1996; 116:237-9.
12. Cohen J. Statistical Power Analysis for the Behavioral Sciences. Second Edition. Lawrence Erlabaum Associates.

## ATTACHMENT 1 – Alternative analyses

In addition to comparing R’s for the different presentations, we will examine the relationship between the expected utility model and what participants decide (Eq 1) with how the information about the risk of heart disease was presented in the following ways:

Fit Eq 1 separately for each group G, and estimate the predicted probability of deciding to take the pill, P(D), for each individual based on this model. The best presentation is the one in which the product moment correlation ?(|A- mean(A)|,|P(D)  D|) is smallest. Since the pseudo-residual |P(D)  D| is a measure of how close each individual decision is to the expected decision for that individual, we would expect this to get smaller with increasing distance of A from its mean, m(A), and to do this faster for the better presentation.

Fit Eq 1 separately for each group G, and estimate the Mahalanobis distance M for each model. M is a measure of the "effectiveness of discrimination" of models such as Eq 1; the greater the value of M, the better the model does at discriminating between groups. The n's are the number choosing the pill or not choosing the pill, the SSs are sums of squares from the regression model, and M can be related to a t-distribution, making comparison between presentations straightforward: if one M is significantly larger than all the others, then that presentation results in a model that "discriminates" better than the models resulting from other presentations.

We will also examine alternative ways of analysing the relationship between the expected utility model and what participants decide (Eq 1) with how the relative importance of the consequences of starting statins is elicited (VAS’s vs LTRS’s).

**ATTACHMENT 2** – Risk presentation

| **Presentation  number** |  | **A. Normal risk presentation** | **B. Increased risk presentation** | **C. Decreased risk presentation** |
| --- | --- | --- | --- | --- |
| **1** | **RRR** | Among those who take the pills, there will be a 33% reduced risk of heart disease during the next 10 years. | Among those who take the pills, there will be a 33% reduced risk of heart disease during the next 10 years. | Among those who take the pills, there will be a 33% reduced risk of heart disease during the next 10 years. |
| **2** | **ARR** | Among those who take the pills, there will be a 2% absolute reduction in the risk of getting heart disease during the next 10 years. | Among those who take the pills, there will be an 8% absolute reduction in the risk of getting heart disease during the next 10 years. | Among those who take the pills, there will be a 0.34% absolute reduction in the risk of getting heart disease during the next 10 years. |
| **3** | **NNT** | Among 50 people who take the pills for the next 10 years, there will be one additional person who will not get heart disease during that time. | Among 13 people who take the pills for the next 10 years, there will be one additional person who will not get heart disease during that time. | Among 294 people who take the pills for the next 10 years, there will be one additional person who will not get heart disease during that time. |
| **4** | **Event rates** | Among those who take the pills, the risk of getting heart disease during the next 10 years will be reduced from 6% to 4%. | Among those who take the pills, the risk of getting heart disease during the next 10 years will be reduced from 24% to 16%. | Among those who take the pills, the risk of getting heart disease during the next 10 years will be reduced from 1% to 0.66%. |
| **5** | **TNT** | Among 50 people that take the pills for the next 10 years, they will swallow a total of 182,500 pills and there will be one additional person who will not get heart disease during that time. | Among 13 people that take the pills for the next 10 years, they will swallow a total of 47,450 pills and there will be one additional person who will not get heart disease during that time. | Among 294 people that take the pills for the next 10 years, they will swallow a total of 1,073,100 pills and there will be one additional person who will not get heart disease during that time. |
| **6** | **Whole numbers** | Among 100 people that do not take the pills, 94 will not get heart disease and 6 will get heart disease during the next 10 years. It is not possible to say whether you would be one of the 94 or one of the 6. Among 100 people that do take the pills, 96 will not get heart disease and 4 will get heart disease during the next 10 years. Again, it is not possible to say whether you would be one of the 96 or one of the 4. | Among 100 people that do not take the pills, 76 will not get heart disease and 24 will get heart disease during the next 10 years. It is not possible to say whether you would be one of the 76 or one of the 24. Among 100 people that do take the pills, 84 will not get heart disease and 16 will get heart disease during the next 10 years. Again, it is not possible to say whether you would be one of the 84 or one of the 16. | Among 1000 people that do not take the pills, 990 will not get heart disease and 10 will get heart disease during the next 10 years. It is not possible to say whether you would be one of the 990 or one of the 10. Among 1000 people that do take the pills, 993 will not get heart disease and 7 will get heart disease during the next 10 years. Again, it is not possible to say whether you would be one of the 993 or one of the 7. |
